# The Phthalic Selenoanhydride Decreases Rat Blood Pressure and Tension of Isolated Mesenteric, Femoral and Renal Arteries

**DOI:** 10.3390/molecules28124826

**Published:** 2023-06-17

**Authors:** Peter Balis, Andrea Berenyiova, Anton Misak, Marian Grman, Zuzana Rostakova, Iveta Waczulikova, Sona Cacanyiova, Enrique Domínguez-Álvarez, Karol Ondrias

**Affiliations:** 1 Institute of Normal and Pathological Physiology, Centre of Experimental Medicine, Slovak Academy of Sciences, 841 04 Bratislava, Slovakia; andrea.berenyiova@savba.sk (A.B.); sona.cacanyiova@savba.sk (S.C.); 2 Institute of Clinical and Translational Research, Biomedical Research Center, Slovak Academy of Sciences, 845 05 Bratislava, Slovakia; anton.misak@savba.sk (A.M.); marian.grman@savba.sk (M.G.); karol.ondrias@savba.sk (K.O.); 3 Institute of Measurement Science, Slovak Academy of Sciences, Dubravska Cesta 9, 841 04 Bratislava, Slovakia; zuzana.rostakova@savba.sk; 4 Faculty of Mathematics, Physics and Informatics, Comenius University, Mlynska Dolina F1, 842 48 Bratislava, Slovakia; iveta.waczulikova@fmph.uniba.sk; 5 Instituto de Química Orgánica General (IQOG), Consejo Superior de Investigaciones Científicas CSIC, Juan de la Cierva 3, 28006 Madrid, Spain; e.dominguez-alvarez@iqog.csic.es

**Keywords:** phthalic selenoanhydride, hemodynamic parameters, vasorelaxation, rats

## Abstract

Phthalic selenoanhydride (R-Se) solved in physiological buffer releases various reactive selenium species including H_2_Se. It is a potential compound for Se supplementation which exerts several biological effects, but its effect on the cardiovascular system is still unknown. Therefore, herein we aimed to study how R-Se affects rat hemodynamic parameters and vasoactive properties in isolated arteries. The right jugular vein of anesthetized Wistar male rats was cannulated for IV administration of R-Se. The arterial pulse waveform (APW) was detected by cannulation of the left carotid artery, enabling the evaluation of 35 parameters. R-Se (1–2 µmol kg^−1^), but not phthalic anhydride or phthalic thioanhydride, transiently modulated most of the APW parameters including a decrease in systolic and diastolic blood pressure, heart rate, dP/dt_max_ relative level, or anacrotic/dicrotic notches, whereas systolic area, dP/dt_min_ delay, dP/dt_d_ delay, anacrotic notch relative level or its delay increased. R-Se (~10–100 µmol L^−1^) significantly decreased the tension of precontracted mesenteric, femoral, and renal arteries, whereas it showed a moderate vasorelaxation effect on thoracic aorta isolated from normotensive Wistar rats. The results imply that R-Se acts on vascular smooth muscle cells, which might underlie the effects of R-Se on the rat hemodynamic parameters.

## 1. Introduction

Selenium (Se) plays a significant role in the regulation of human health. As an essential trace element, the levels of Se in the organism are dependent on the diet [1,2]. For instance, when there is a lack of Se in the diet, selenocompounds are mostly used as food supplements [3,4,5,6,7,8,9]. Both selenocompounds and the Se atom present in the form of selenocysteine at the active sites of selenoproteins participate in physiological processes and have a protective role towards several diseases, including cancer, diabetes, neurodegenerative and cardiovascular disorders, inflammation, or infections [2,3,4,5,6,7,8,9,10,11,12,13,14,15,16,17,18]. However, chronic overconsumption of Se may lead to intoxication with compromised functions of the kidney, immune, and reproductive system as well as to the development of cancer, cardiovascular, and liver diseases [2,19].

**Scheme 1:**
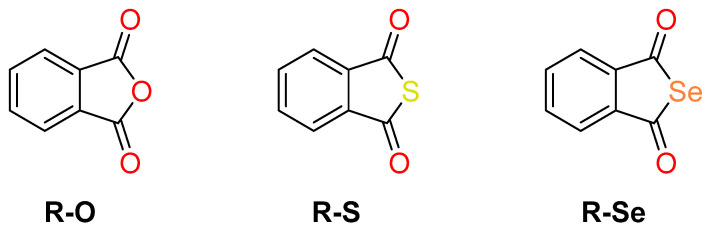
Chemical structures of the three evaluated compounds: phthalic anhydride (R-O) and its sulfur (phthalic thioanhydride, R-S) and selenium (phthalic selenoanhydride, R-Se) isosteres.

Therefore, new Se-compounds are studied as suitable Se-donors to have direct beneficial effects on diseases [18,20,21,22]. In this context, data previously reported by the group showed that phthalic selenoanhydride (R-Se, Scheme 1), the Se isostere of phthalic anhydride (R-O), has a noteworthy chemopreventive, cytostatic, cytotoxic, free radical scavenging, apoptotic, antiviral, antimicrobial, antibiofilm, and multidrug-resistance (MDR) reversing activity compared with the poor or null activity of its sulfur (phthalic thioanhydride, R-S) and oxygen isosteres (phthalic anhydride, R-O). R-Se has been recently suggested to be a potential candidate for a safe anticancer drug [23]. These facts point to the relevance of the Se atom of R-Se to the biological activities observed [24,25,26,27,28,29].

In addition, Se has a marked influence on the cardiovascular system, as, for example, urinary Se concentration is negatively associated with systolic and diastolic blood pressure (BP), implying that Se exerts a protective action against an increased BP [30]. This fact also points out that Se deficiency might be a risk factor for high BP development [31]. The activation of the antioxidant enzyme glutathione peroxidase (GPx) using Se as a cofactor may partly account for the observed negative association between Se concentration and BP [32]. ROS-related processes such as lipid peroxidation, atherosclerotic plaque formation, and platelet aggregation can be reduced by an enhancement of GPx activity [30,33,34,35]. Alternatively, high Se concentrations, among older adults, may be significantly associated with cardiometabolic risk factors [36,37,38]. Recent data have confirmed that serum Se concentrations have a U-shaped relationship with systolic BP and pulse pressure and with cardiovascular mortality in hypertensive patients, suggesting that sufficient Se levels may contribute to BP control and hypertension prevention. Meanwhile, too low or too high a concentration of serum Se might be associated with hypertension diseases [30,31,36,39,40,41].

An increased artery tension, which decreases blood flow, is the basis of many diseases and death [42,43,44]. Therefore, compounds that reduce arterial tension are interesting for experimental studies to find novel derivatives with a potential application in medical practice. Reported data suggest that Se affects arterial functions; however, its effects are not fully documented. As a source of Se, sodium selenite supplementation was found to enhance acetylcholine-induced relaxation of isolated rat aortic rings, but a direct application of selenite on the rings had no effect [45]. In the rat femoral artery vasospasm model, intraperitoneal sodium selenite morphometrically prevents the development of peripheral vasospasms [46]. Concentration of serum Se was reported to be positively associated with arterial stiffness and BP in humans [47]. 

We have previously shown that selenite in the presence of H_2_S, was able to modify the tension of the precontracted aortic rings isolated from normotensive rats [48]. Therefore, we have assumed that the direct interaction of Se or Se-compounds with the cardiovascular regulatory system should not be underestimated. The proposed mechanism suggested to explain the activity of R-Se is the release from R-Se, which would act as a prodrug [24,26]. In our previous study, a complex pattern of various reactive Se species, including H_2_Se, was observed after the fragmentation of R-Se in a 50% methanol/H_2_O solution [29]. We hypothesized that R-Se could directly influence BP and tensions of isolated arteries.

The vasoactive effect of novel pharmacological agents is of great interest, especially regarding arterial contribution to vascular resistance and the pressure control, which mainly include small blood vessels mesenteric, femoral, and renal arteries, with diameters between 200 and 500 µm [49,50]. The main bioactive molecule revealing significant vasorelaxant effect in blood vessels is the nitric oxide (NO) produced by the endothelium. In animal models, where there is an optimal state of health, the role of NO is intact, and pharmacologically-administered drugs reflects the acute real state of relaxation-constriction responses. 

In the present work, R-Se was used as the donor of reactive Se species including H_2_Se to study their direct effect on the cardiovascular system. The effect of intravenous (IV) bolus administration of R-Se on rat hemodynamic parameters and tension of isolated rat mesenteric, femoral, and renal arteries was studied. Additionally, we bring new results elucidating the impact of novel R-Se on the endothelial and/or vascular smooth muscle cells. 

## 2. Results

### 2.1. R-Se, but Not R-S or R-O Modulates Arterial Pulse Waveform

**Figure 1:**
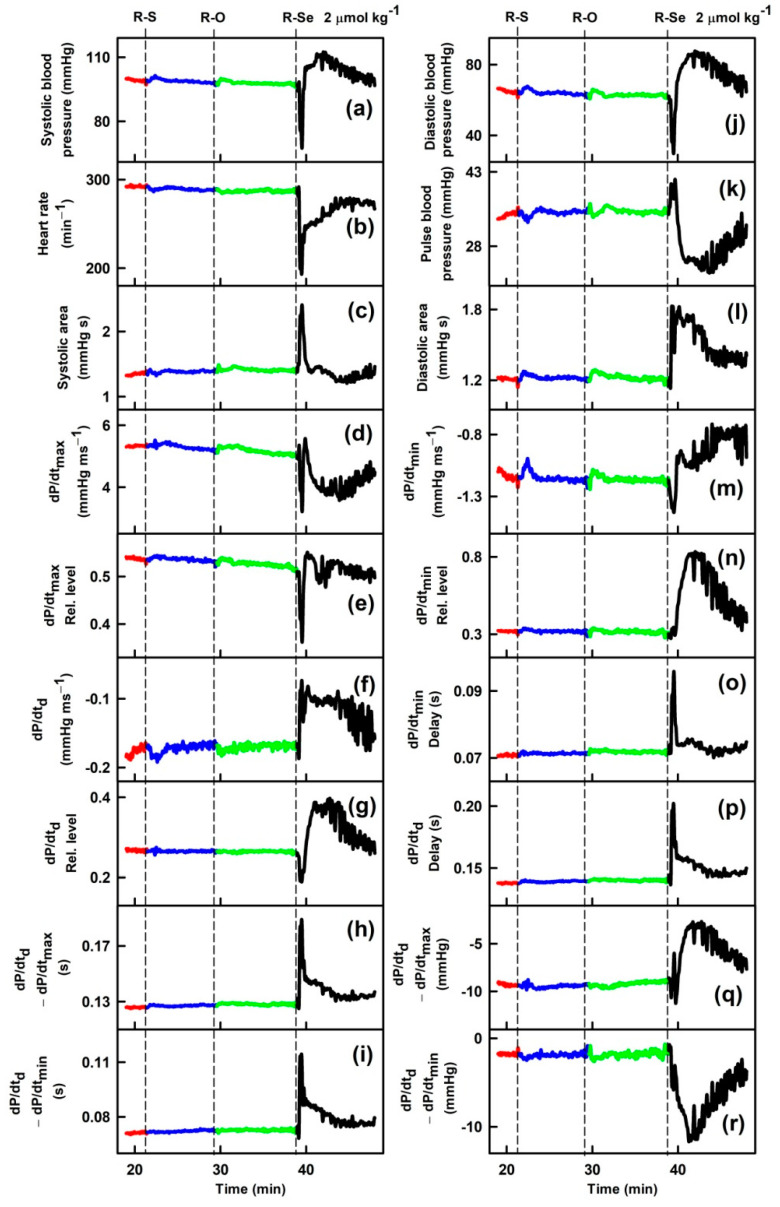

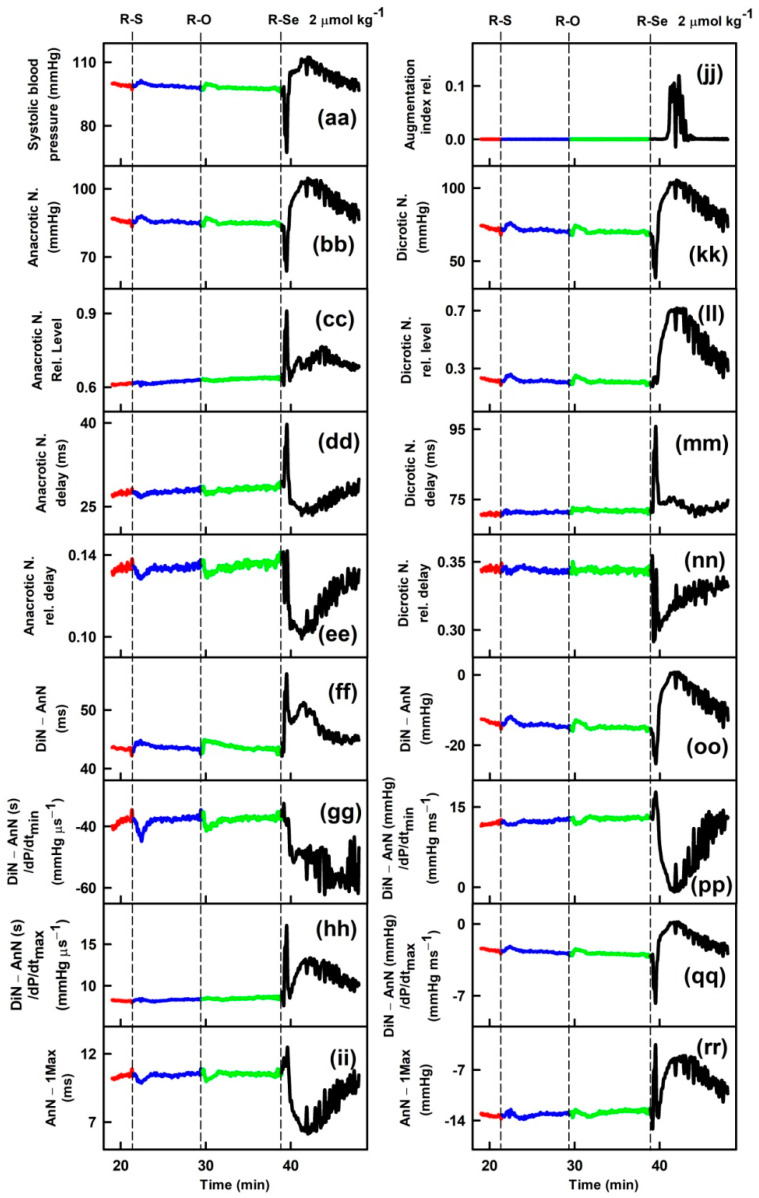
Time-dependent changes in 35 rat arterial pulse waveform parameters (APW-Ps) of anaesthetized rats in control (red) and after intravenous administration of 2 µmol kg^−1^ phthalic anhydride (R-O, green) and its phthalic thioanhydride (R-S, blue) and phthalic selenoanhydride (R-Se, black) isosteres. Abbreviations, definitions, and units of measured APWs from arterial pulse waveform (APW) are as explained previously [51,52] and briefly in Supplementary Material Figure S1.

**Figure 2:**
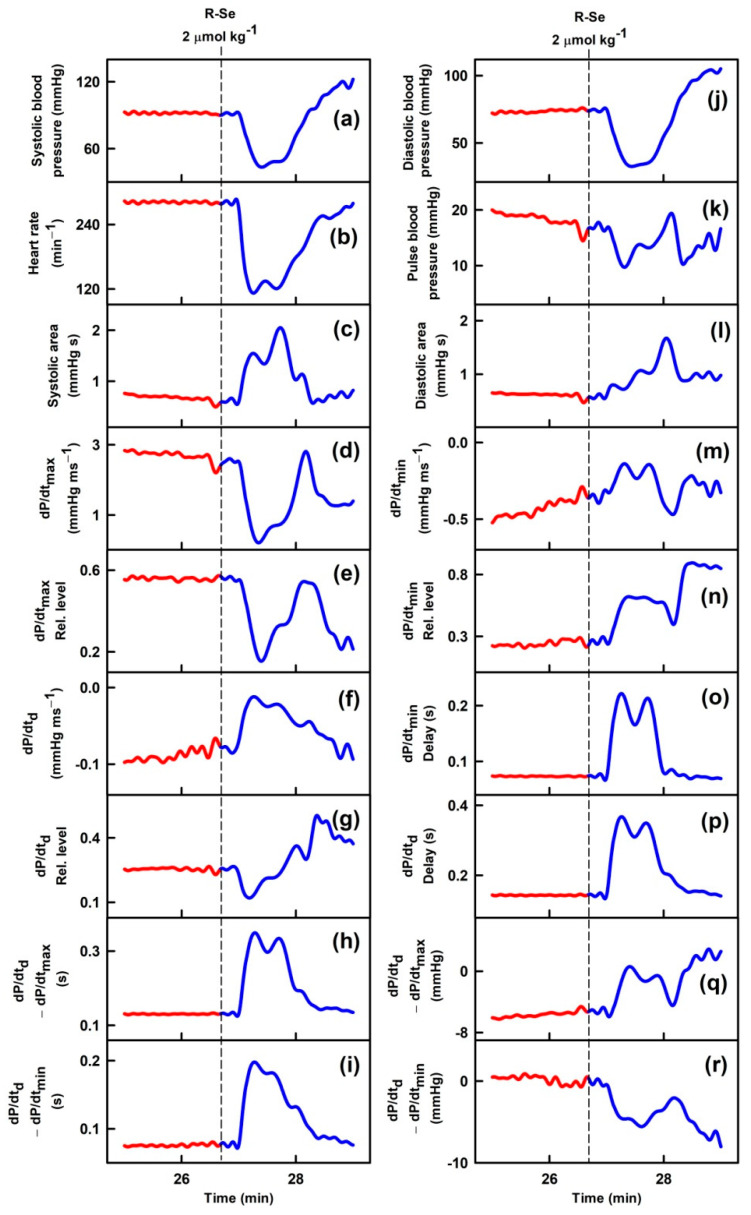

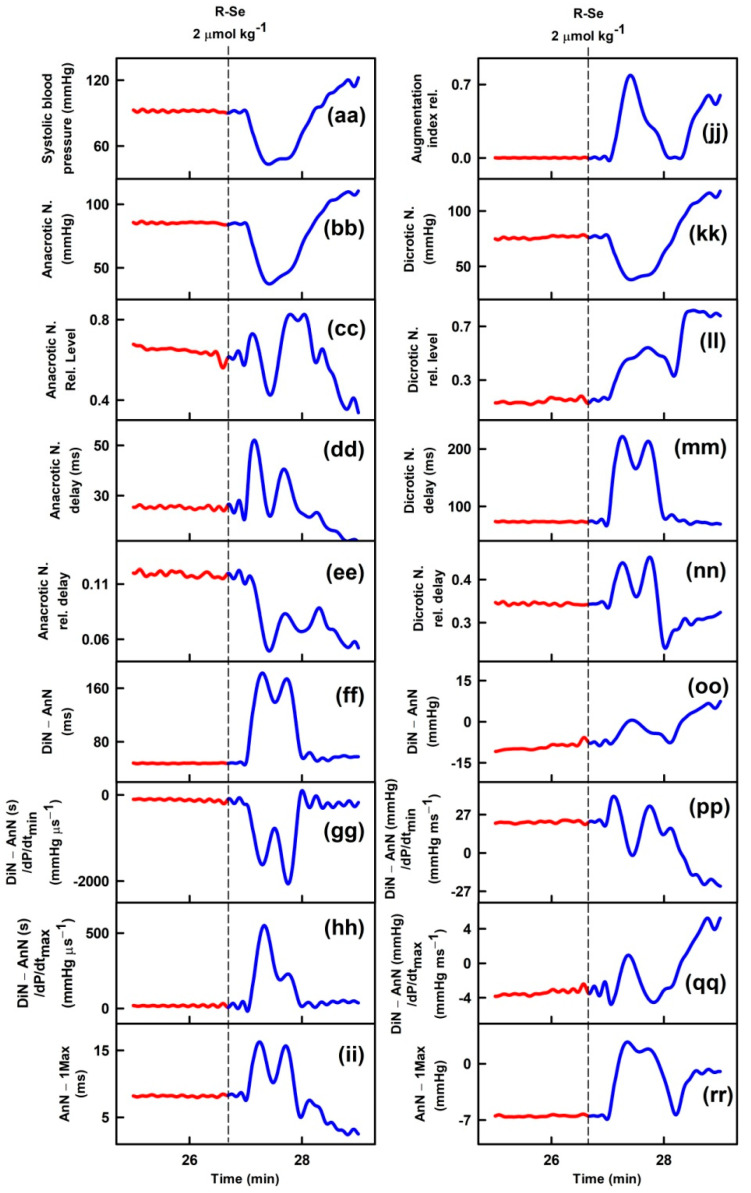
Time-dependent changes of 35 APW-Ps of anaesthetized rats before (red) and after intravenous bolus (15 s) administration of 2 µmol kg^−1^ R-Se (blue). Vertical dash lines indicate the start of R-Se administration. Abbreviations, definitions, and units of measured APW-Ps from the APW are explained in [51,52] and briefly in Figure S1.

Parameters of rat arterial pulse waveform (APW-Ps) were evaluated after IV bolus administration of the tested anhydride-containing compounds R-O, R-S and R-Se. Administration of R-S or R-O at 1 or 2 µmol kg^−1^ (*n* = 5) had negligible effects on all 35 APW-Ps (Figure 1). However, administration of R-Se (1 or 2 µmol kg^−1^, *n* = 2 + 7) transiently (for approximately 1 min) altered most APW parameters followed by changes very likely attributable to the body’s response to a decrease in the BP (Figure 1). The details of the time-dependent changes in APWs (Figure 2 and Figures S2–S9) revealed that changes in some parameters showed simple transient behavior, for example, systolic or diastolic BP, heart rate, dP/dt_max_, dP/dt_d_, augmentation index, or anacrotic and dicrotic notches, while other parameters did not follow changes in the systolic BP, for example, pulse BP, systolic and diastolic area, or anacrotic and dicrotic notches rel. levels or delays. Qualitative changes were mostly reproducible, but quantitative data were scattered. Therefore, we summed together the qualitative increase or decrease in the APW-Ps after R-Se administration of 1 and 2 µmol kg^−1^ (Table 1). The same qualitative changes were observed in 15 of 35 APW-Ps in all nine rats, including a decrease in the systolic and diastolic BP, heart rate, dP/dt_max_ relative level, or anacrotic and dicrotic notches, whereas systolic area, dP/dt_min_ delay, dP/dt_d_ delay, anacrotic notch relative level, or delay increased. In eight of nine rats, the same qualitative changes were observed in four APW-Ps including a decrease in dP/dt_d_ relative level or an increase in the diastolic area. In seven out of nine rats, the same qualitative changes were observed in five APW-Ps including an increase in dP/dt_d_ or pulse BP, and a decrease in dP/dt_max_, or anacrotic notch relative delay. In the remaining 11 APW parameters, qualitative changes were less pronounced and therefore they were difficult to be distinguished. After R-Se (1 and 2 µmol kg^−1^) administration, the systolic BP decreased by 28 ± 14% (*n* = 9, ±SD).

### 2.2. R-Se Decreased Tension of Isolated Mesenteric, Femoral and Renal Arteries

**Figure 3:**
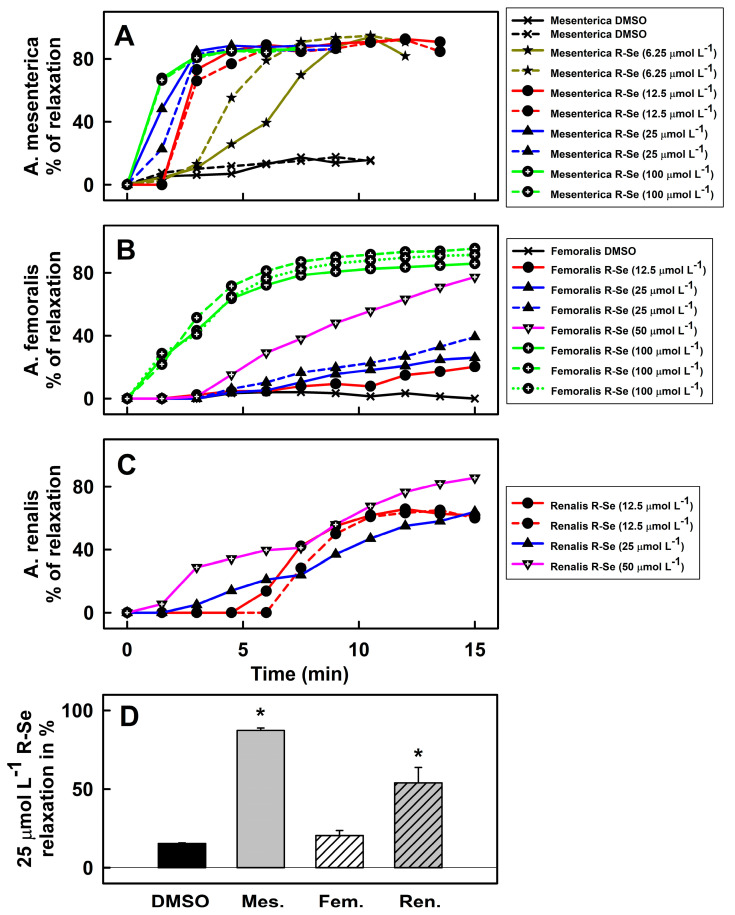
Effect of R-Se on the vascular reactivity of the mesenteric artery ((**A**) *A. mesenterica*), femoral artery ((**B**) *A. femoralis*), and renal artery ((**C**) *A. renalis*). The vasoactive effects of R-Se on the *A. mesenterica* (**A**) were pre-contracted with NA (10 µmol L^−1^), *A. femoralis* (**B**) and *A. renalis* (**C**) with Ser (1 µmol L^−1^). The relaxing effect of R-Se was observed for 14−15 min. Comparison of the relaxation effect of R-Se (25 µmol L^−1^) on mesenteric (Mes.), femoral (Fem.) and renal (Ren.) artery measured at 10.5th min (**D**). * *p* < 0.05 vs. DMSO (one-way analysis of variance followed by Tukey-Kramer test for multiple comparisons).

To know which part of arterial tree contributed to the observed BP decrease, the effects exerted by R-Se on isolated mesenteric, femoral, and renal arteries, as well as on the thoracic aorta were studied. Noradrenaline (NA, 10 µmol L^−1^) increased tension of the mesenteric artery and cumulative concentration of acetylcholine (1 nmol L^−1^–10 µmol L^−1^) decrease it, confirming the proper function of a contraction-relaxation mechanism (Figure S10). NA was used to increase tension again and subsequent application of R-Se (6.25; 12.5; 25; and 100 µmol L^−1^) relaxed mesenteric artery. After washing out of R-Se, NA was applied again to induce contraction (Figure S10). R-Se relaxed mesenteric artery in concentration dependent manner (Figure 3A) and its effects prevailed after washed out of R-Se, so the next contraction was significantly lower (Figure S11). The addition of DMSO alone did not relax the artery (Figure 3A). Similar results were obtained when studying the effect of R-Se on the femoral (Figure 3B) and renal arteries (Figure 3C). In these cases, first the tension of arteries was induced by serotonin (1 µmol L^−1^). Then, a control cumulative concentration of acetylcholine relaxed them, before inducing the arterial tension by serotonin again. The subsequent application of R-Se (12.5–100 µmol L^−1^) relaxed the arteries. R-Se significantly relaxed all arteries ≥14 min after application. Effect of R-Se on femoral artery was less pronounced than that on mesenteric and renal arteries (Figure 3D). The overall effect of R-Se (25 µmol L^−1^) on the relaxation of arteries (measured in the 10.5th minute post administration of R-Se) was highly significant (*p* = 0.0019, one-way analysis of variance, Figure 3D).

### 2.3. R-Se Had a Minor Effect on the Tension of the Isolated Thoracic Aorta

**Figure 4:**
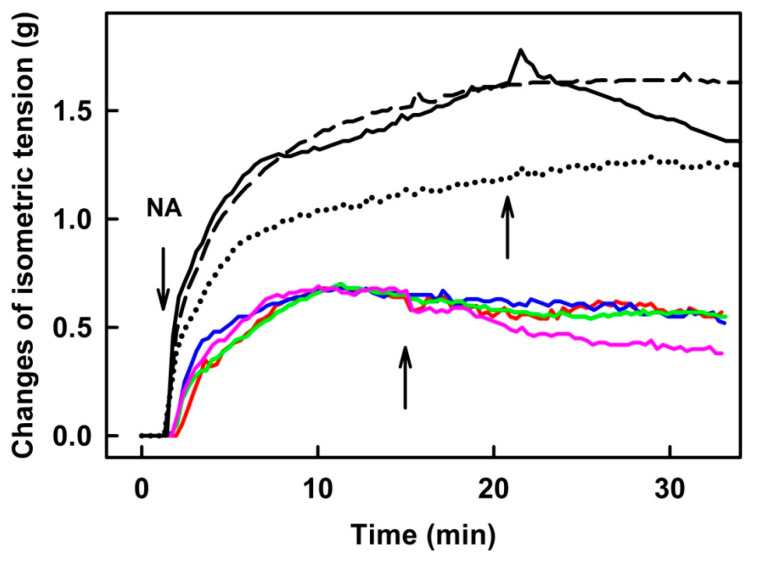
The original records of time-dependent changes in noradrenaline (NA, 0.1 µmol L^−1^—color curves; 1 µmol L^−1^—black curves; left arrow)—increased tension of isolated thoracic aorta and the subsequent application of 25 (dotted), 50 (dash), 100 (solid black line), 200 (red, blue, green) and 300 µmol L^−1^ R-Se (pink). The middle (for colors) and right arrows (for blacks) indicate R-Se application.

NA (0.1 and 1 µmol L^−1^) increased the tension of the isolated thoracic aorta. A subsequent application of 25 or 50 µmol L^−1^ of R-Se had no vasoactive effect on the precontracted thoracic aorta. When higher concentrations were tested (100–300 µmol L^−1^), they triggered a mild vasorelaxation (11–38%) (Figure 4). DMSO ≤ 0.6% *v*/*v* had no effects on the aorta tonus.

## 3. Discussion

According to the lines of evidence presented in previous works, R-Se has the capacity to release different reactive Se species (RSeS). Among them, the release of hydrogen selenide (H_2_Se) can be highlighted, as this RSeS is able to interact with specific cellular compounds and proteins, having different ex vivo or in vivo effects [24,25,26,27,28,53]. According to our knowledge, this is the first study of the direct effect of an intravenously-administrated Se-compound that is capable of releasing reactive Se species (RSeS), including H_2_Se, on APW-Ps in an animal model. Due to rats under deep anaesthesia used in our study, some control APW-Ps as systolic BP or heart rate were lower than in unanaesthetized rats [54,55]. The qualitative effects of R-Se on APW-Ps were mostly reproducible, but the quantitative data were scattered (Figure 1 and Figure 2). It might be assumed that this could be caused by different rat anaesthetic conditions and/or unequal concentrations of the products formed due to slightly different times between the dilution of R-Se from DMSO solution with saline and its administration. Time-dependent changes of UV-Vis spectra of Na_2_Se in phosphate buffer were observed indicating a reaction of Na_2_Se with the components of the buffer [29].

Qualitative effects of R-Se on APW-Ps were reproducible and they were probably directly related to the Se atom, as no relevant effects were displayed by its sulfur (phthalic thioanhydride, R-S), or oxygen (phthalic anhydride, R-O) isosteres (Figure 1). Transient effects of R-Se are similar to those found for H_2_S-donor Na_2_S, and NO-donor S-nitrosoglutathione [52,56]. It is assumed that the transient effect of R-Se could be a consequence of the instability in the blood of the active product(s) released from R-Se and/or their effective elimination by the kidney. These possibilities are supported by ex vivo experiments, in which non-transient relaxation effects of R-Se on isolated arteries were found (Figure 3). Some of the time-dependent biphasic changes in APW-Ps (e.g., increase in systolic BP, Figure 1a) after the transient effect of R-Se are not yet understood; however, they could be associated with a sympathetic reflex response reported for well-known vasoactive substances such as endothelin, urotensin, and apelin [57,58,59]. Qualitative similar transient biphasic changes in systolic BP were observed after IV administration of H_2_S-donor Na_2_S [56]. Similar effects, but much less pronounced, were observed after the administration of sodium selenite (Na_2_SO_3_) [48]. Under the same experimental conditions, the capacity of R-Se (1 and 2 µmol kg^−1^) to decrease the systolic BP (by 28 ± 14%, means ± SD) was significantly higher than that reported for sodium selenite at a higher micromolar concentration (5 µmol kg^−1^): only a 1.3% BP systolic decrease was determined for the inorganic salt [48].

It was hypothesized that it might be possible to characterize the cardiovascular system status in diverse pathophysiological conditions along with the effects of pharmacological drugs through the analysis of the detailed shape of APW [60,61,62,63,64,65,66,67,68]. Thirty-five APW-Ps were defined to look for patterns of changes in APW under different cardiovascular conditions [51,52]. R-Se influenced most of the 35 APW-Ps. However, we cannot know with the available data whether the observed changes in these parameters, indicative of cardiovascular effects, can be attributed to the Se, to the R-Se compound, or to the reactive Se species that could be released from R-Se.

We focused on experimental methods ex vivo on the conduit aorta and small vessels, to know to what extent they participated in the observed decrease in BP after R-Se administration. The results indicate that all used vascular segments relaxed in presence of R-Se in the concentration-dependent manner. The most sensitive vascular segments among the ones used were the small resistant mesenteric arteries. Since the mesenteric arteries are part of the splanchnic circulation, which receives about a quarter of the total cardiac output [69], marked vasodilation in these arteries (even at low R-Se concentrations of 6.25 µmol L^−1^) could correlate with a decrease in systemic BP. Consistent with the resistance of mesenteric artery vasodilation, our data demonstrate that acute R-Se treatment indeed lowers systemic BP. When the vascular type changes from small resistant to large conduit, the strength of the relaxation effect of R-Se decreases. After R-Se application, the subsequent precontraction of the vascular segment was partially or significantly inhibited (Figures S10 and S11), and it may indicate prolonged interaction with constriction/relaxation pathways.

Based on previously reported scientific works, it was assumed that the vasoprotective effects of various Se-donors were a consequence of their potential participation in the reduction of the production of superoxide radicals and an in the increase in NO basal levels [70,71]. The treatment with H_2_Se enables a potential reversion of the H_2_O_2_-induced oxidative stress through the regulation of the glutathione peroxidase (GPx1) and thioredoxin reductase (TrxR2) selenoproteins. The pretreatment with H_2_Se provides a protective effect against H_2_O_2_-induced oxidative stress, cell death, and cardiac hypertrophy [72]. 

In the cardiovascular system, NO is produced primarily in endothelial cells and subsequently affects smooth muscle cells evoking relaxation. Se supplements were shown to improve the bioavailability of NO in various models of induced endothelial dysfunction [45,73]. For this reason, we may assume that exogenous R-Se can positively influence the function of endothelial cells as described in other works. However, R-Se compound at higher concentration evoked only mild vasorelaxant effect in thoracic aorta. Since the relaxation in thoracic aorta is mainly NO-mediated [74], these results are aligned with the suggestion that R-Se could relax the vessel wall by a mechanism excluding NO derived from the endothelium. There are other endothelium-derived relaxing factors (EDRFs), such as prostacyclin or endothelium-derived hyperpolarizing factors (EDHFs), which could participate in the R-Se related relaxation. EDHFs have been described as one of the principal mediators of endothelium-dependent vasorelaxation in small resistance arteries in normotensive animals [75]. Endothelium-independnet mechanizms, such as blockade of Ca^2+^ channels cannot be excluded [76]. Other additional experiments are needed to figure out which of the listed vasorelaxant mechanizms are included.

Our findings that R-Se transiently decreased rat systolic and diastolic BP and modulated several other hemodynamic parameters and that it significantly decreased the tension of the isolated arteries point to the direct effect of R-Se to relax arteries leading to BP reduction. Cardiovascular diseases, as the leading cause of death worldwide, include disorders related to BP and function of the heart or blood vessels [77]. Since an increased tension of the arteries, which causes a decrease in blood flow, is the basis of many diseases and death [42,43,44,77], R-Se potency to relax arteries is interesting for pharmacological studies potentially leading to application in medical practice.

## 4. Materials and Methods

### 4.1. Chemistry

Herein, three chalcogen phthalic anhydrides have been evaluated: the phthalic selenoanhydride (R-Se), the phthalic thioanhydride (R-S), and the phthalic anhydride (R-O), whose structure has been shown in the Scheme 1 at the introduction [24]. R-O was commercially available (Merck, Taufkirchen, Germany), whereas R-Se was obtained through a synthetic route that enables the synthesis of R-Se from lithium aluminum hydride, selenium phthloyl chloride, and sulfuric acid, as described in prior works [24]. R-S was synthesized following the same route, but using sulfur instead of selenium. Tiletamine + zolazepam (Zoletil 100) was acquired from Virbac (Carros, France), and the anaesthetic xylazine was obtained from Merck (Schnelldorf, Germany). All other chemicals required for the performance of this study (DMSO, NaCl, KCl, NaHCO_3_, MgSO_4_, KH_2_PO_4_, CaCl_2_, Na_2_EDTA, glucose) were purchased from Sigma-Aldrich.

### 4.2. Guide for the Use and Care of Experimental Animals

Adult male normotensive Wistar rats (*n* = 14; 320 ± 30 g) were obtained from the Department of Toxicology and Laboratory Animal Breeding at Dobra Voda, Slovak Academy of Sciences, Slovakia. The rats were housed under a 12 h-light–12 h-dark cycle at a constant humidity and temperature (45–65% and 20–22 °C, respectively) with free access to standard laboratory rat chow and drinking water ad libitum. The veterinary nursing care was provided by the Central Animal Housing Facility of Pavilion of Medical Sciences (registration number SK UCH 01017). The procedures counted with the approval of the corresponding Slovakian committee (the State Veterinary and Food Administration of the Slovak Republic; C.k. Ro 3123/17-221) taking into account the European Parliament guidelines given at the 2010/63/EU Directive. The procurement of animals, the husbandry, and the performance of the experiments were in accordance with the protocols and guidelines of the ”European Convention for the Protection of Vertebrate Animals used for Experimental and other Scientific Purposes” (Council of Europe No 123, Strasbourg 1985). All the performed experiments were carried out according to the guidelines established by the animal welfare committee of the Biomedical Research Center Slovak Academy of Sciences, and Centre of Experimental Medicine of the Slovak Academy of Sciences, Bratislava, and conformed to the principles and regulations as described in the editorial by Grundy [78]. All animal experiments complied with the ARRIVE guidelines.

### 4.3. APW Measurement and Data Evaluation

The method used was described in our previous studies [51,52]. To ensure the effect of anaesthesia lasts for 60 min, rats (*n* = 9) were anaesthetized with Zoletil 100 (tiletamine + zolazepam, 80 mg kg^−1^, IP) and xylazine (5 mg kg^−1^, IP). The left common carotid artery (*arteria carotis communis*) was cannulated to insert the fiber optic microcatheter pressure transducer FISO LS 2F connected to the FISO Series signal conditioner embedded in the EVO chassis (Harvard Apparatus, Holliston, MA, USA) [79]. The recorded analogue APW signal was filtered by a low-pass filter at 1 kHz, digitalized at 10 kHz and stored on a computer. Fresh stock solutions of R-S, R-O and R-Se (50 mmol L^−1^) were prepared daily using DMSO as solvent. Just before the administration, the stock solutions were diluted with 0.9% saline to reach a final concentration of 1 or 2 µmol kg^−1^ in the rats. R-S, R-O, or R-Se were administered into the right jugular vein (500 µL kg^−1^) over a 15 s period, approximately 30–40 min after the anaesthetic application. The following time scale was used: After the chirurgical operation of the rat under anesthesia, we waited 15–20 min for stabilization of BP, then bolus of R-Se (or R-S or R-O) was administered, and effect of the compound was recorded for 10 min. When BP was still stable as in the control, in some cases for comparison, a second administration of the same or different compounds was administered, and the effect was recorded for 10 min. For the evaluation of the R-Se effects, the first administration was taken only. Ten points (**a**–**j**) of APW, marked in Figure S1, were analyzed, from which 35 APW parameters (APW-Ps) were calculated. Some APW-Ps are commonly used as hemodynamic parameters and others were defined to detect more changes in APW that could be attributed to the influence of a studied substance. The definition and abbreviation of the 35 APW-P parameters are described in the supplementary material (Figure S1), and a more detailed description is available in a previous study [51]. For a better visual comparison, plots (a) and (aa) representing systolic BP in the figures are the same. The plot of the relative augmentation index (jj) was not able to determine when the highest point at APW (Figure S1) was “**c**” and not “**f**” and was set to zero [51]. The animals were under anesthesia throughout the duration of the experiment at 37 °C and were euthanized with an overdose of Zoletil/xylazine administered via the jugular vein at the end of the surgical procedure. Thirty-five APW-Ps were defined to look for patterns of changes in APW under different cardiovascular conditions [51,52], and some of them are commonly used as hemodynamic parameters.

### 4.4. Measurement of the Vasoactive Effect of R-Se

#### 4.4.1. Vascular Tissue Collection

Normotensive Wistar rats (*n* = 5) were killed by decapitation after a brief anesthetization with CO_2_, and the thoracic aorta (TA), mesenteric, femoral, and renal arteries were isolated as described in our previous studies [80,81,82]. Briefly, aortic, mesenteric, femoral, and renal vascular tissue were carefully cleaned from surrounding connective and adipose tissue and cut to the required lengths according to the type of device used to measure vascular reactivity.

#### 4.4.2. Functional Study of Rat-Isolated Mesenteric, Femoral and Renal Arteries

Approximately 1.6 mm cut-off long vascular segments of the small mesenteric (first-order branches of the inferior mesenteric artery), femoral, and renal arteries were mounted as ring-shaped preparations in the Mulvany-Halpern style small vessel wire myograph chamber (Dual Wire Myograph System 410A, DMT A/S, Aarhus, Denmark) to determine the vascular reactivity at isometric conditions in the modified physiological salt solution (PSS, in mmol L^−1^: NaCl 118.99, KCl 4.69, NaHCO_3_ 25, MgSO_4_ 1.17, KH_2_PO_4_ 1.18, CaCl_2_ 2.5, Na_2_EDTA 0.03, glucose 5.5, pH 7.4) [83]. After mounting, the arteries were maintained in oxygenated PSS (a mixture of 95% O_2_, and 5% CO_2_ at a stabilized temperature of 37 °C). 

**Scheme 2:**
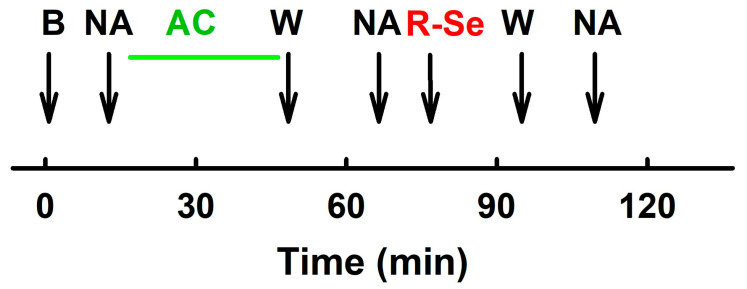
After the stabilization period (**B**) the vascular segments were contracted with **NA** (10 µmol L^−1^, mesenteric artery) or Ser (1 µmol L^−1^, femoral and renal arteries) and relaxed with gradual addition of acetylcholine (**AC**, green line, 1 nmol L^−1^–10 µmol L^−1^) to test the contractility of the segment. The solution was washed out (**W**). After the next stabilization period, the segments were again pre-contracted with **NA** (or Ser) and the effects of **R-Se** (stock solution, 50 mmol L^−1^ in DMSO) were added. R-Se was washed out (**W**) and **NA** (or Ser) were applied to contract the vascular segment again.

The experiment protocol was modified according to [82], briefly (Scheme 2): normalization and stabilization of the vascular segment took place as described previously. After the stabilization period, vessel segments were contracted with serotonin (Ser, 1 µmol L^−1^, femoral, and renal arteries) or NA (10 µmol L^−1^, mesenteric artery) and relaxed with acetylcholine (1 nmol L^−1^−10 µmol L^−1^). The solution was washed out. After the next stabilization period, the segments were again pre-contracted with a constrictor substance, and the effects of R-Se (stock solution, 50 mmol L^−1^ in DMSO) were studied. Subsequently, the functionality of the arteries was tested by changes in the pre-contraction of the vascular segment. Effect of R-Se on segments of arteries was statistically evaluated using analysis of variance and non-linear regression methods [84]. A *p*-value < 0.05 was considered statistically significant. Statistical analyses were performed using GraphPad Prism 9.0 (GraphPad Software Inc., San Diego, CA, USA).

#### 4.4.3. Functional Study of the Isolated Thoracic Aorta

Vascular segments of the thoracic aorta (approximately 5 mm long) were vertically fixed between two stainless wire triangles and placed into a 20 mL organ bath with Krebs solution (in mmol L^−1^: 118 NaCL, 5 KCL, 25 NaHCO_3_, 1.2 MgSO_4_, 1.2 KH_2_PO_4_, 2.5 CaCl_2_, 11 glucose, 0.032 CaNa_2_EDTA). The solution was oxygenated with 95% O_2_ and 5% CO_2_ and kept at 37 °C. The upper triangles were connected to isometric tension sensors (FSG-01, MDE, Budapest, Hungary), and changes in tension were registered by an AD converter (MDE, Budapest, Hungary). Changes in isometric tension were registered by SPEL Advanced Kymograph (MDE, Budapest, Hungary) software. A resting tension of 1 g was applied to each ring and maintained throughout a 45 to 60-min equilibration period until stress relaxation no longer occurred. The vasoactive effect of R-Se was tested on aortic rings pre-contracted by noradrenaline (NA, 0.1 or 1 µmol L^−1^). 

## 5. Conclusions

In conclusion, we found that the phthalic selenoanhydride (R-Se) transiently decreased rat systolic and diastolic blood pressure. In addition, this selenocompound modulated several other hemodynamic parameters and it significantly decreased the tension of the isolated arteries. Overall, our data point to the direct effect of R-Se to relax arteries leading to blood pressure reduction. Since cardiovascular diseases are the leading cause of death worldwide and include disorders related to blood pressure and function of the heart or blood vessels, these cardiovascular properties of R-Se may be interesting for application studies potentially leading to future use in medical practice. 

## Figures and Tables

**Table 1:** The sum of changes in 35 arterial pulse waveform parameters (APW-Ps) in nine anaesthetized rats after intravenous administration of 1 and 2 µmol kg^−1^ phthalic selenoanhydride (R-Se). Arrow indicates transient increase or decrease in particular APW-Ps after R-Se administration. The number indicates how many times the increase or decrease was observed out of nine experiments. Symbol ”~“ indicates a not clearly resolved effect of R-Se. Data were evaluated visually from Figure 2 and Figures S2–S9.

APW-Ps	Variation	APW-Ps	Variation
(a) Systolic blood pressure	↓ = 9	(aa) Systolic blood pressure	↓ = 9
(b) Heart rate	↓ = 9	(bb) Anacrotic notch	↓ = 9
(c) Systolic area	↑ = 9	(cc) Anacrotic notch relative level	↑ = 9
(d) dP/dt_max_	↓ = 7	(dd) Anacrotic notch delay	↑ = 9
(e) dP/dt_max_ relative level	↓ = 9	(ee) Anacrotic notch relative delay	↓ = 7
(f) dP/dt_d_	↑ = 7	(ff) [Dicrotic notch (DiN) in s] − [Anacrotic notch (AnN) in s]	↑ = 9
(g) dP/dt_d_ relative level	↓ = 8	(gg) [(DiN − AnN) in s]/[dP/dt_min_ in mmHg µs^−1^]	~
(h) dP/dt_d_ − dP/dt_max_	↑ = 9	(hh) [(DiN − AnN) in s]/[dP/dt_max_ in mmHg µs^−1^]	↑ = 8
(i) dP/dt_d_ − dP/dt_min_	↑ = 9	(ii) [AnN in ms] − [1Max in ms]	↑ = 7
(j) Diastolic blood pressure	↓ = 9	(jj) Augmentation index relative	~
(k) Pulse BP	↑ = 7	(kk) Dicrotic notch	↓ = 9
(l) Diastolic area	↑ = 8	(ll) Dicrotic notch relative level	~
(m) dP/dt_min_	↓ = 6	(mm) Dicrotic notch delay	↑ = 9
(n) dP/dt_min_ relative level	~	(nn) Dicrotic notch relative delay	~
(o) dP/dt_min_ delay	↑ = 9	(oo) [DiN in mmHg] − [AnN in mmHg]	↓ = 7
(p) dP/dt_d_ delay	↑ = 9	(pp) [(DiN − AnN) in mmHg]/[dP/dt_min_ in mmHg ms^−1^]	↑ = 6
(q) dP/dt_d_ − dP/dt_max_	~	(qq) [(DiN − AnN) in mmHg]/[dP/dt_max_ in mmHg ms^−1^]	↓ = 6
(r) dP/dt_d_ − dP/dt_min_	~	(rr) [AnN in mmHg] − [1Max in mmHg]	↑ = 8

## Data Availability

Most of the data are presented in the main text and in the supplement to the article. All original records are available from Peter Balis.

## Supplementary Materials

The following supporting information can be downloaded at: https://www.mdpi.com/article/10.3390/molecules28124826/s1, Figure S1. The left common carotid artery pulse waveform (APW) in the anesthetized rat. Control APW with marked ten points a–j. Figures S2–S9. Time-dependent changes of 35 APW-Ps of anaesthetized rat before and after IV bolus administration of R-Se. Figure S10. Original recording of the vascular reactivity of the mesenteric artery and effects of noradrenaline, acetylcholine and R-Se. Figure S11. Effects of noradrenaline, serotonin and R-Se on tension of the mesenteric, renal and femoral arteries.
