# Supplementary material for: The Phthalic Selenoanhydride Decreases Rat Blood Pressure and Tension of Isolated Mesenteric, Femoral and Renal Arteries
Source: Molecules. 2023 Jun 17;28(12):4826. doi: 10.3390/molecules28124826 (PMC10304488; doi:10.3390/molecules28124826)
Supplement: Supplementary file 1 [file molecules-28-04826-s001.zip › molecules-2402985-supplementary.pdf]

Supplementary material

to

The Phthalic Selenoanhydride Decreases Rat Blood Pressure and Tension of Isolated Mesenteric, Femoral and Renal Arteries

Peter Balis [1,*], Andrea Berenyiova [1], Anton Misak [2], Marian Grman [2], Zuzana Rostakova [3], Iveta Waczulikova [4], Sona Cacanyiova [1], Enrique Domínguez-Álvarez [5] and Karol Ondrias [2]

1 Institute of Normal and Pathological Physiology, Centre of Experimental Medicine, Slovak Academy of Sciences, 841 04 Bratislava, Slovakia; andrea.berenyiova@savba.sk (A.B.); sona.cacanyiova@savba.sk (S.C.)
2 Institute of Clinical and Translational Research, Biomedical Research Center, Slovak Academy of Sciences, 845 05 Bratislava, Slovakia; anton.misak@savba.sk (A.M.); marian.grman@savba.sk (M.G.); karol@ondrias.sk (K.O.)
3 Institute of Measurement Science, Slovak Academy of Sciences, Dubravska cesta 9, 841 04 Bratislava, Slovakia; zuzana.rostakova@savba.sk
4 Faculty of Mathematics, Physics and Informatics, Comenius University, Mlynska dolina F1, 842 48 Bratislava, Slovakia; iveta.waczulikova@fmph.uniba.sk
5 Instituto de Química Orgánica General (IQOG), Consejo Superior de Investigaciones Científicas CSIC , Juan de la Cierva 3, 28006 Madrid, Spain; e.dominguez-alvarez@iqog.csic.es
* Correspondence: peter.balis@savba.sk

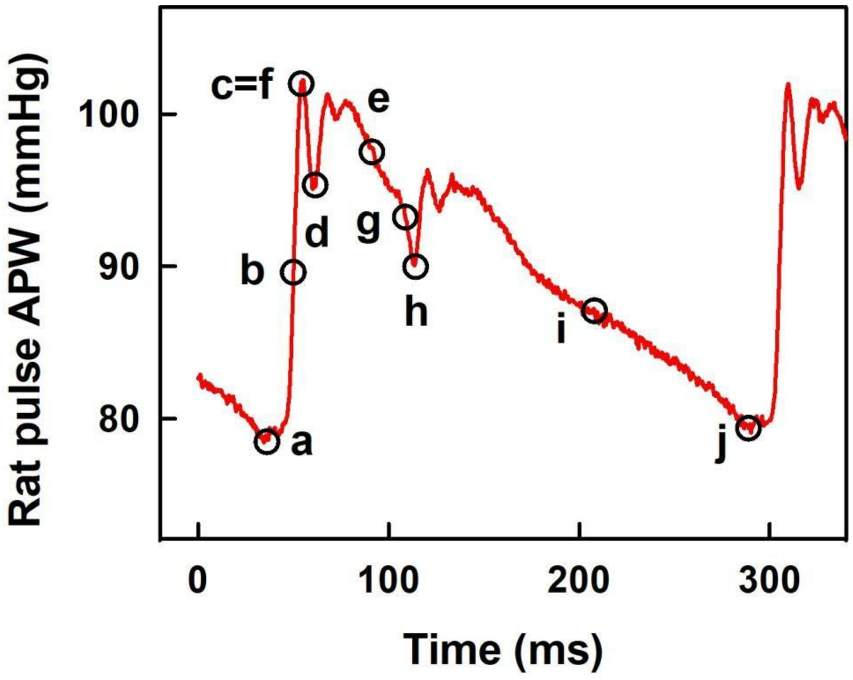

Figure S1. The left common carotid artery pulse waveform (APW) in the anesthetized rat. Control APW with marked ten points **a** - **j** (black circles).

Description of 35 APW parameters (APW-Ps) from APW.

Ten points **a - j** (in bold letters) are from Figure S1 and they mark the values of BP and time that are used to define (calculate) specific APW-Ps

(a) Systolic blood pressure in mmHg; point **c** or **f, point with maximal mmHg**.

(b) Heart rate in min^{-1}; 60 / (**j** – **a**); (**j** – **a**) represents time interval between **a** and **j**, **a** and **j** are two reference points to diastolic BP value.

(c) Systolic area in mmHg s; integral BP of **a** to **h**; **h** refers to BP at the dicrotic notch (dicrotic BP).

(d) dP/dt_{max} in mmHg ms^{-1}; maximum derivative at the point **b**; P is BP in mmHg.

(e) dP/dt_{max} relative level; relative level (RL) of point **b**; (**b** – **a**) / (**c** (or **f**) – **a**) in mmHg/mmHg (dimensionless).

(f) dP/dt_{d} in mmHg ms^{-1}; negative derivative at the point **i**; the point **i** is the BP in the middle of the time interval between **h** and **j**.

(g) dP/dt_{d} relative level, relative level of point **i**; (**i** – **a**) / (**c** (or **f**) – **a**) in mmHg/mmHg (dimensionless).

(h) dP/dt_{d} – dP/dt_{max} in s; time interval between **b** and **i**, dP/dt_{d} – dP/dt_{max} = (**i** – **b**).

(i) dP/dt_{d} – dP/dt_{min} in s; time interval between **g** and **i**, dP/dt_{d} – dP/dt_{min} = (**i** – **g**); dP/dt_{min} is maximum negative derivative at the point **g**.

(j) Diastolic blood pressure in mmHg; the point **a** or **j.**

(k) Pulse BP in mmHg; (**c** – **a**) or (**f** – **a**).

(l) Diastolic area in mmHg s; integral BP of **h** to **j**.

(m) dP/dt_{min} in mmHg ms^{-1}; dP/dt_{min} is maximum negative derivative at the point **g**.

(n) dP/dt_{min} relative level, relative level of point **g**; (**g** – **a**) / (**c** (or **f**) – **a**) in mmHg/mmHg (dimensionless).

(o) dP/dt_{min} delay in s; delay in s of point **g**; (**g** – **a**) time interval between **a** and **g**.

(p) dP/dt_{d} delay in s; delay in s of point **i**; (**i** – **a**) time interval between **a** and **i**.

(q) dP/dt_{d} – dP/dt_{max} in mmHg; (**i** – **b**) BP difference between **b** and **i**.

(r) dP/dt_{d} – dP/dt_{min} in mmHg; (**i** – **g**) BP difference between **g** and **i**.

(aa) Systolic blood pressure in mmHg; point **c** or **f**. Plot (aa) is the same as (a).

(bb) Anacrotic notch in mmHg; BP at the point **d**.

(cc) Anacrotic notch relative level; relative level of point **d**; (**d** – **a**) / (**c** (or **f**) – **a**) in mmHg/mmHg (dimensionless).

(dd) Anacrotic notch delay in ms; delay in ms of point **d**; (**d** – **a**) time interval between **a** and **d**.

(ee) Anacrotic notch relative delay; relative delay (RD) of point **d**; (**d** – **a**) / (**j** – **a**) in ms/ms (dimensionless)

(ff) [Dicrotic notch (DiN) in s] – [Anacrotic notch (AnN) in s] in s; (**h** – **d**) time interval between **d** and **h**.

(gg) [(DiN – AnN) in s] / [dP/dt_{min} in mmHg μs^{-1}] in s/mmHg μs^{-1}; (**h** – **d**) / **g**.

(hh) [(DiN – AnN) in s] / [dP/dt_{max} in mmHg μs^{-1}] in s/mmHg μs^{-1}; (**h** – **d**) / **b**.

(ii) [AnN in ms] – [1Max (point **c** or the 1st. maximum) in ms] in ms; (**d** – **c**) time interval between **c** and **d**.

(jj) Augmentation index relative; (**f** – **c**) / (**f** – **a**) in mmHg/mmHg (dimensionless)**.

(kk) Dicrotic notch in mmHg; BP at the point **h**.

(ll) Dicrotic notch relative level; relative level of point **h**; (**h** – **a**) / (**c** (or **f**) – **a**) in mmHg/mmHg (dimensionless).

(mm) Dicrotic notch delay in ms, delay in ms of point **h**; (**h** – **a**).time interval between **a** and **h**.

(nn) Dicrotic notch relative delay; relative delay of point **h**; (**h** – **a**) / (**j** – **a**); in ms/ms (dimensionless)

(oo) [DiN in mmHg] – [AnN in mmHg] in mmHg; (**h** – **d**) BP difference between **d** and **h**;

(pp) [(DiN – AnN) in mmHg] / [dP/dt_{min} in mmHg ms^{-1}] in mmHg/mmHg ms^{-1}; (**h** – **d**) / **g**;

(qq) [(DiN – AnN) in mmHg] / [dP/dt_{max} in mmHg ms^{-1}] in mmHg/mmHg ms^{-1}; (**h** – **d**) / **b**.

(rr) [AnN in mmHg] – [1Max (point **c** or the 1st. maximum) in mmHg] in mmHg; (**d** – **c**) BP difference between **c** and **d**.

*Units in plots (gg), (hh), (pp) and (qq) are informative only.

The plot of augmentation index relative (jj**) was not possible to determine in cases when the highest point at APW (Figure S1A) was "c" and not "f" and it was set to zero [1,2]

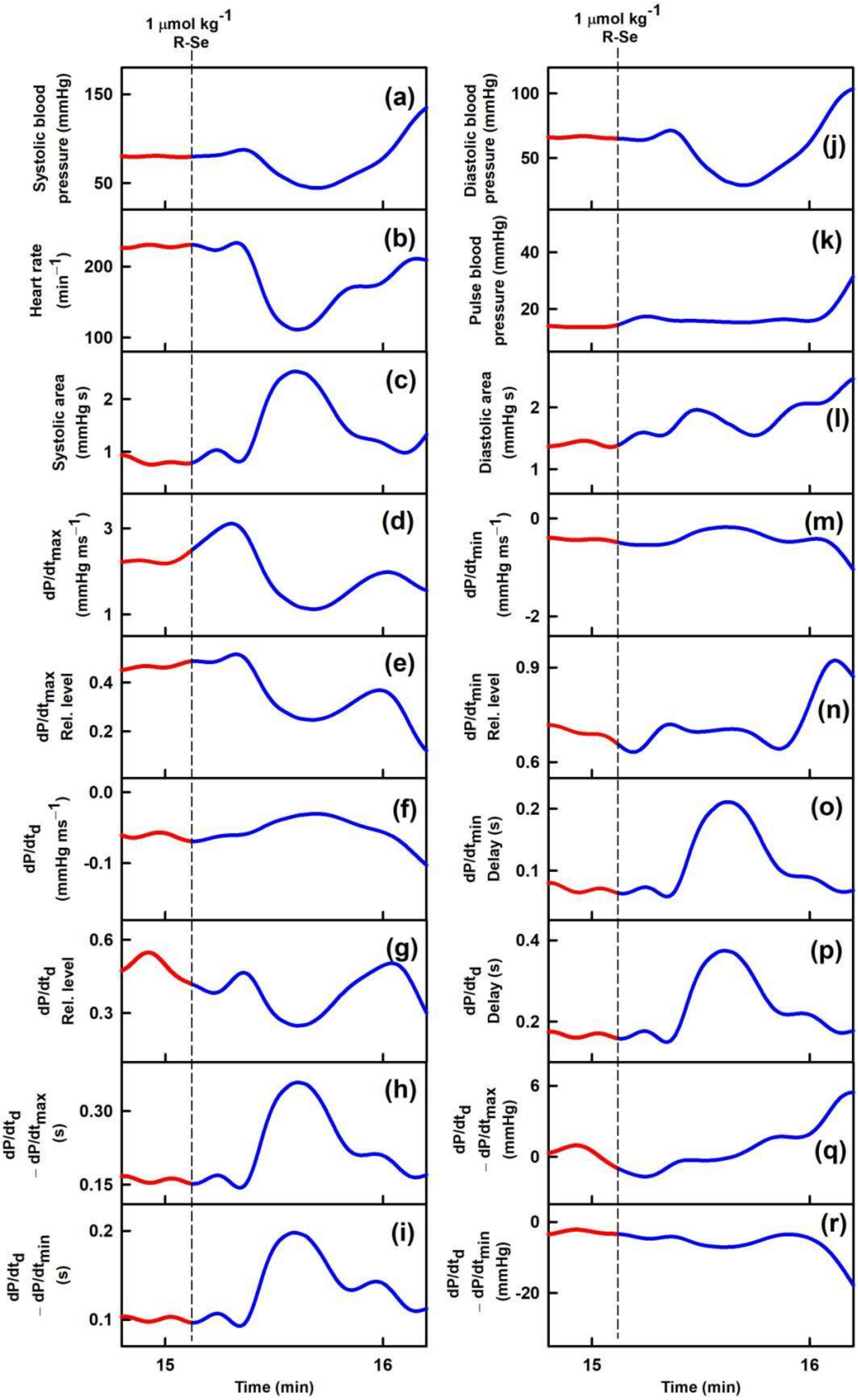
1 μmol kg-1
R-Se
Systolic blood pressure (mmHg)
Heart rate (min−1)
Systolic area (mmHg s)
dP/dtmax (mmHg ms−1)
dP/dtmax Rel. level
dP/dtd (mmHg ms−1)
dP/dtd Rel. level
dP/dtd − dP/dtmax (s)
dP/dtd − dP/dtmin (s)
Diastolic blood pressure (mmHg)
Pulse blood pressure (mmHg)
Diastolic area (mmHg s)
dP/dtmin (mmHg ms−1)
dP/dtmin Rel. level
dP/dtmin Delay (s)
dP/dtd Delay (s)
dP/dtd − dP/dtmax (mmHg)
dP/dtd − dP/dtmin (mmHg)
(a)
(b)
(c)
(d)
(e)
(f)
(g)
(h)
(i)
(j)
(k)
(l)
(m)
(n)
(o)
(p)
(q)
(r)
Time (min)
15
16

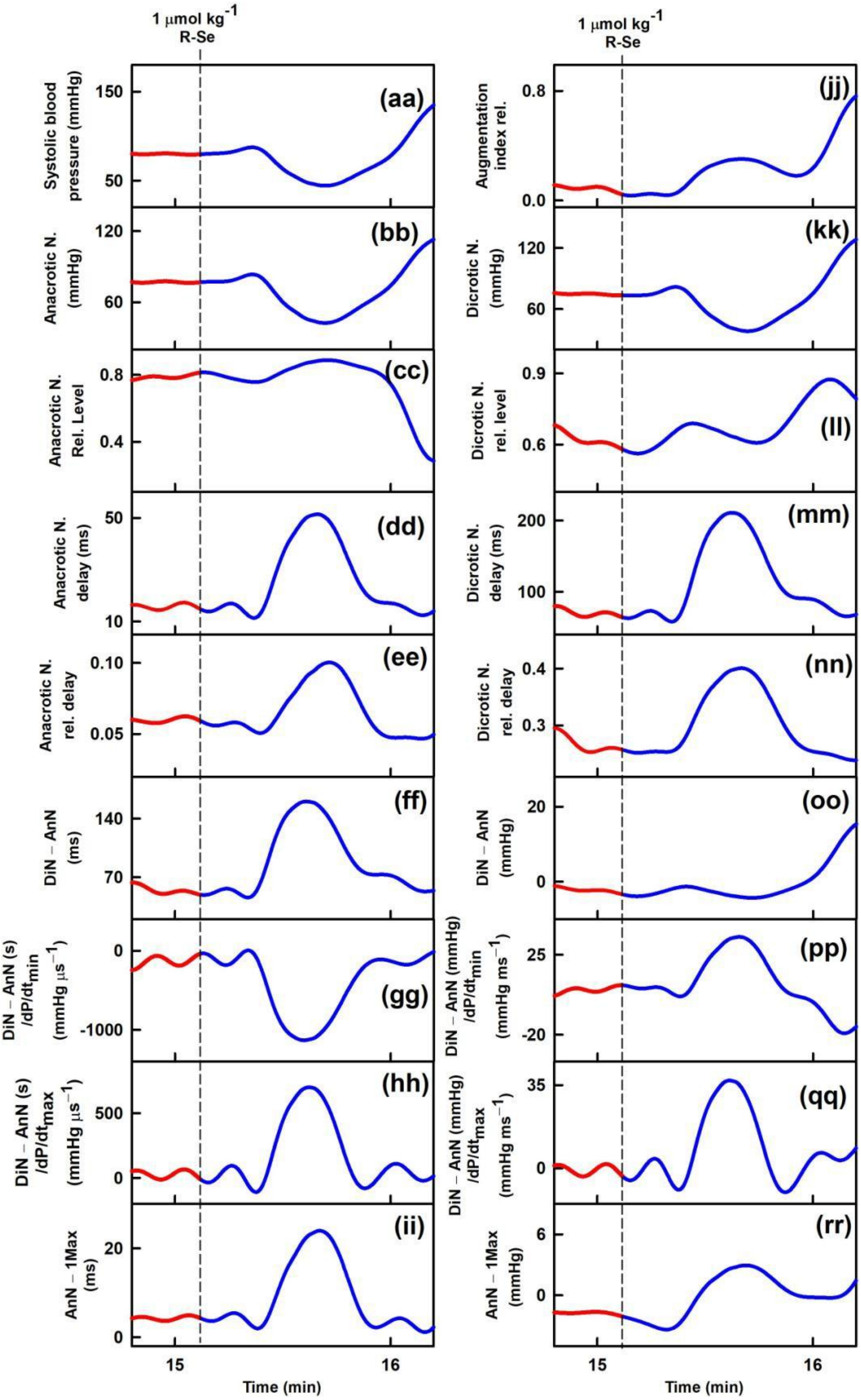

Figure S2. Time-dependent changes of 35 APW-Ps of anaesthetized rat before (red) and after IV bolus (15 s) administration of 1 μmol kg^{-1} phthalic selenoanhydride (R-Se, blue). Vertical dash lines indicate start of R-Se administration. Definitions, units and abbreviations of APW-Ps evaluated from the APW are explained in [1,2] and briefly in Figure S1.

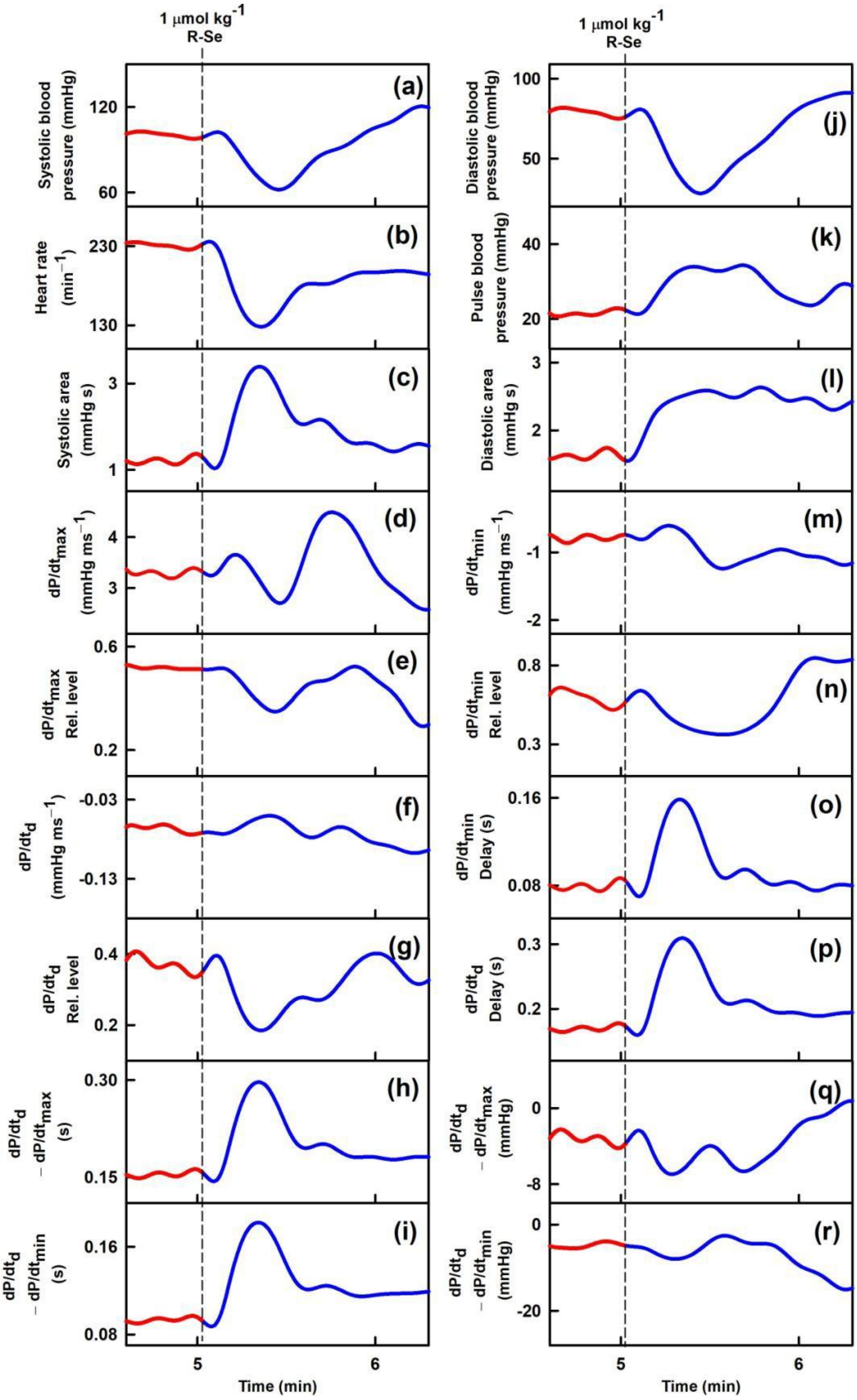

1 μmol kg-1
R-Se
Systolic blood pressure (mmHg)
Heart rate (min-1)
Systolic area (mmHg s)
dP/dtmax (mmHg ms-1)
dP/dtmax Rel. level
dP/dtd (mmHg ms-1)
dP/dtd Rel. level
dP/dtd – dP/dtmax (s)
dP/dtd – dP/dtmin (s)
Diastolic blood pressure (mmHg)
Pulse blood pressure (mmHg)
Diastolic area (mmHg s)
dP/dtmin (mmHg ms-1)
dP/dtmin Rel. level
dP/dtmin Delay (s)
dP/dtd Delay (s)
dP/dtd – dP/dtmax (mmHg)
dP/dtd – dP/dtmin (mmHg)
Time (min)
(a)
(b)
(c)
(d)
(e)
(f)
(g)
(h)
(i)
(j)
(k)
(l)
(m)
(n)
(o)
(p)
(q)
(r)

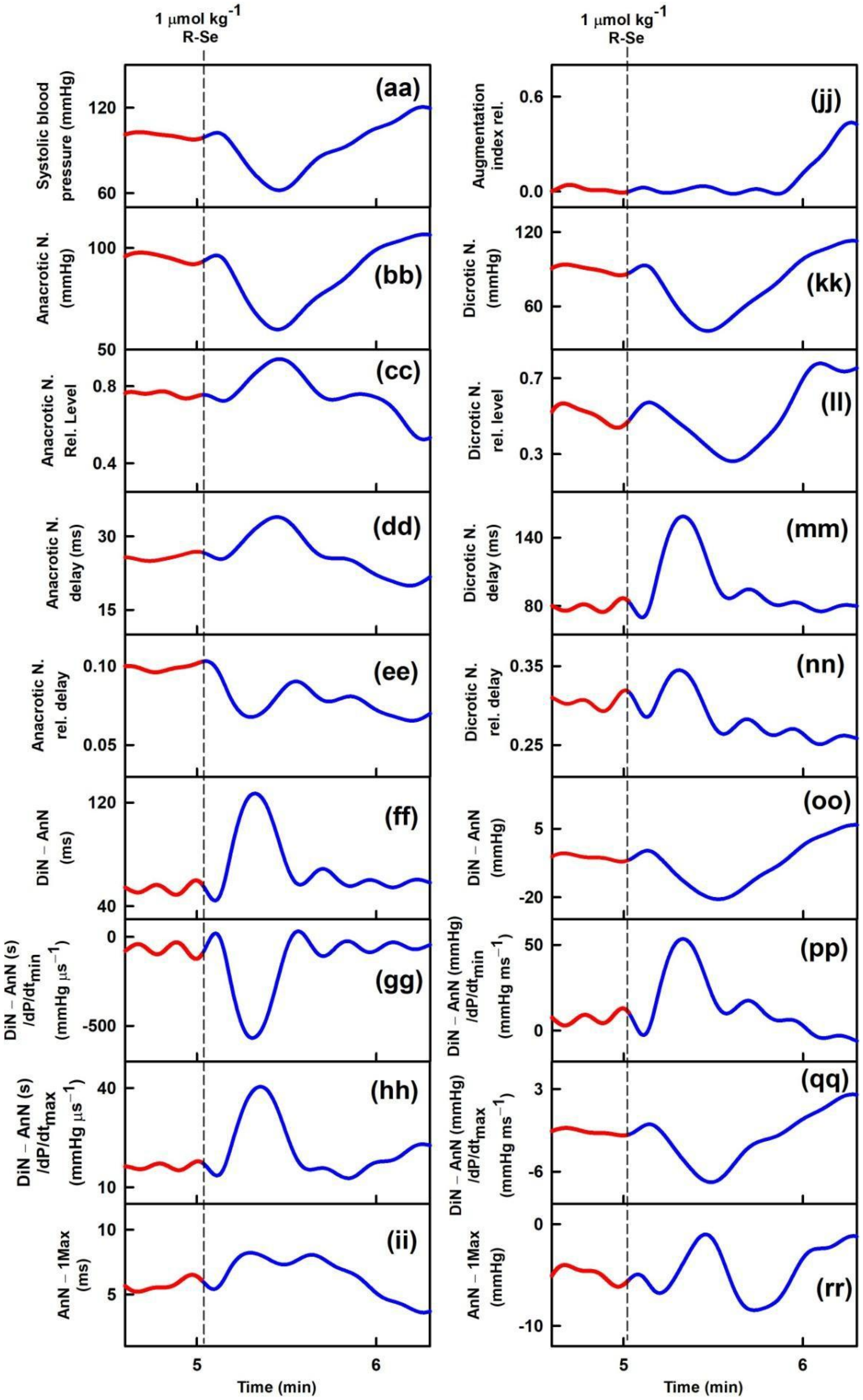

Figure S3. Time-dependent changes of 35 APW-Ps of anaesthetized rat before (red) and after IVIV bolus (15 s) administration of 1 μmol kg^{-1} R-Se (blue). Vertical dash lines indicate start of R-Se administration. Definitions, units and abbreviations of APW-Ps evaluated from the APW are explained in [1,2] and briefly in Figure S1.

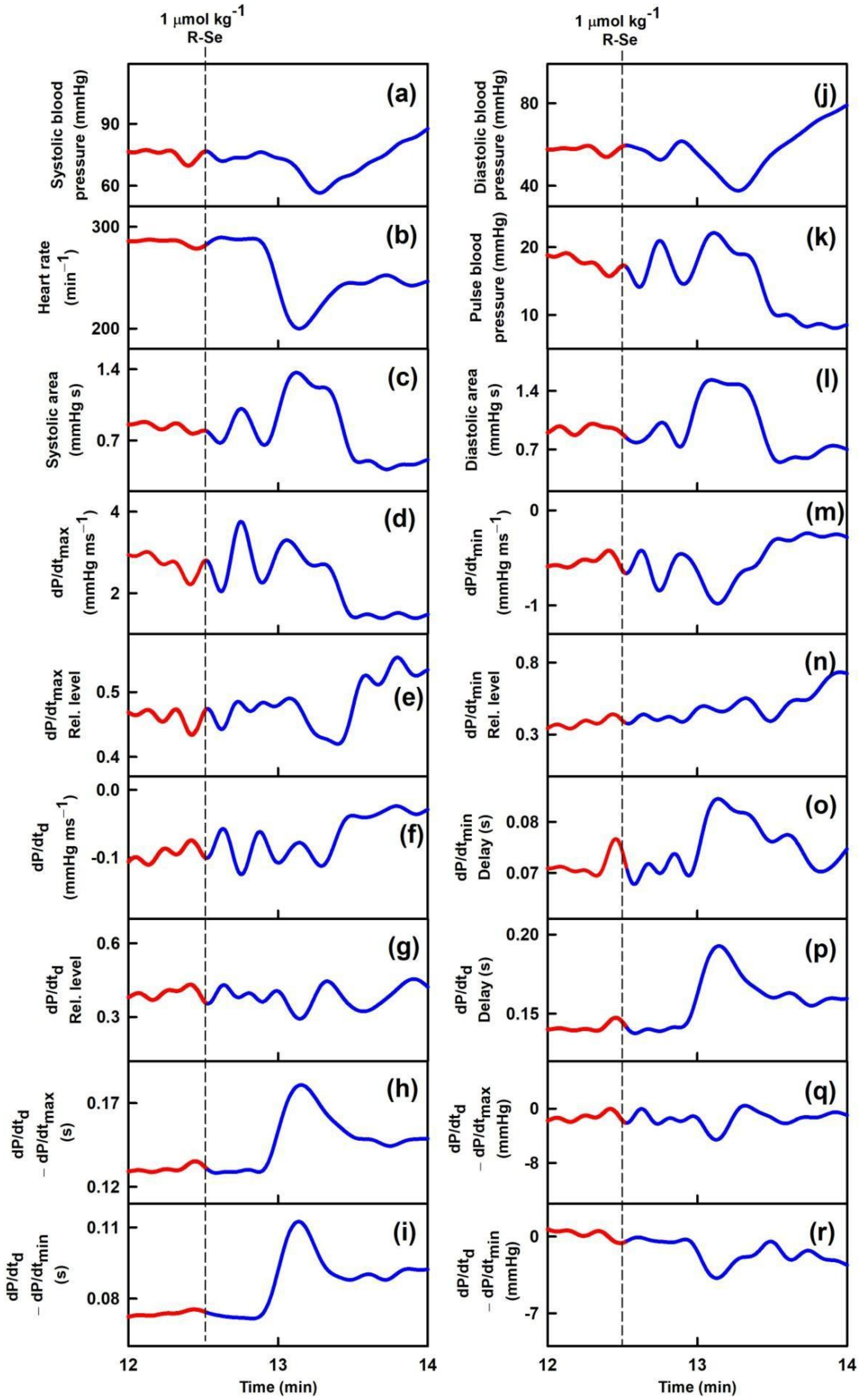
1 μmol kg-1
R-Se
Systolic blood pressure (mmHg)
(a)
Heart rate (min-1)
(b)
Systolic area (mmHg s)
(c)
dP/dtmax (mmHg ms-1)
(d)
dP/dtmax Rel. level
(e)
dP/dtd (mmHg ms-1)
(f)
dP/dtd Rel. level
(g)
dP/dtd – dP/dtmax (s)
(h)
dP/dtd – dP/dtmin (s)
(i)
Time (min)
Diastolic blood pressure (mmHg)
(j)
Pulse blood pressure (mmHg)
(k)
Diastolic area (mmHg s)
(l)
dP/dtmin (mmHg ms-1)
(m)
dP/dtmin Rel. level
(n)
dP/dtmin Delay (s)
(o)
dP/dtd Delay (s)
(p)
dP/dtd – dP/dtmax (mmHg)
(q)
dP/dtd – dP/dtmin (mmHg)
(r)
Time (min)

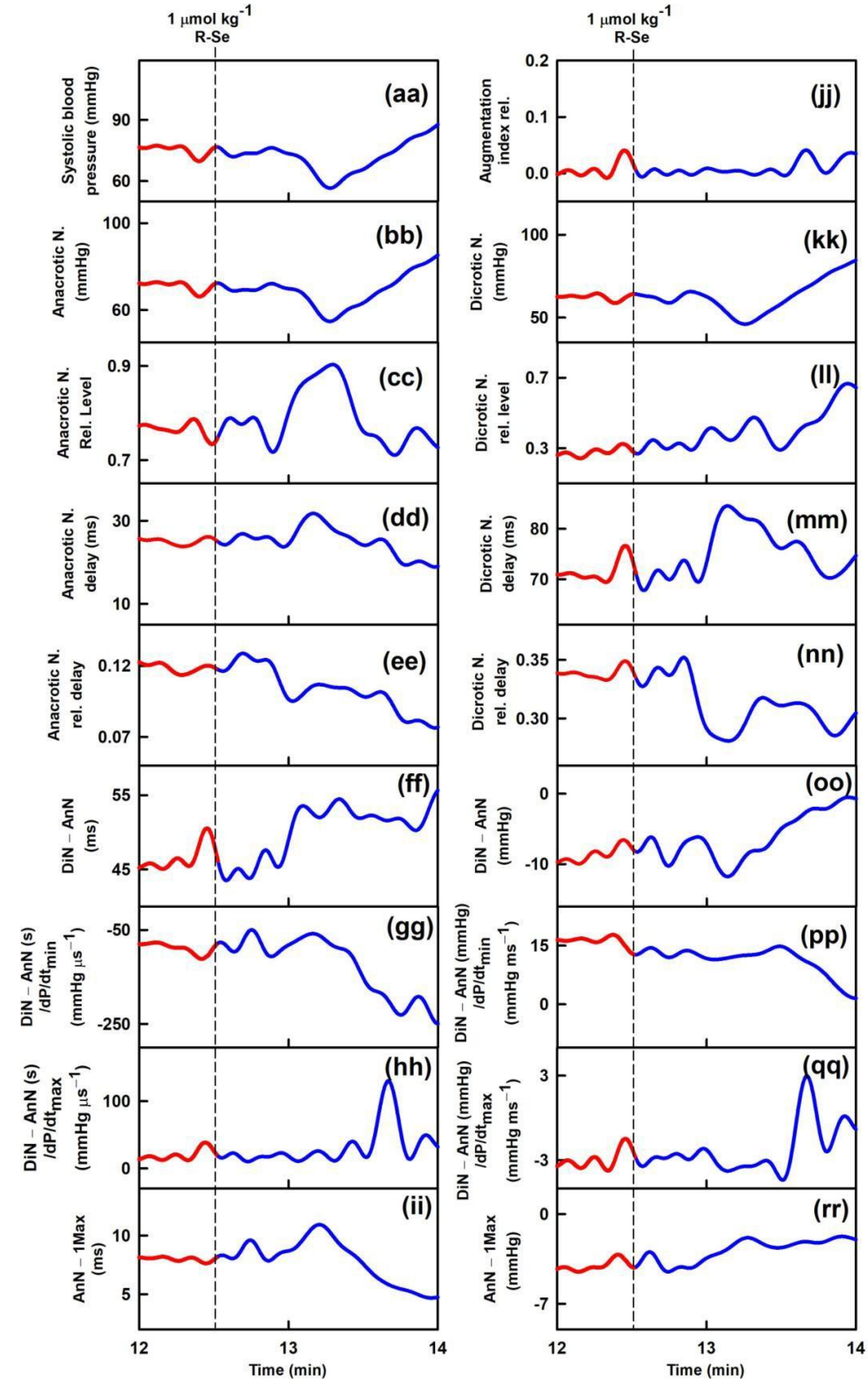

Figure S4. Time-dependent changes of 35 APW-Ps of anaesthetized rat before (red) and after IV bolus (15 s) administration of 1 μmol kg^{-1} R-Se (blue). Vertical dash lines indicate start of R-Se administration. Definitions, units and abbreviations of APW-Ps evaluated from the APW are explained in [1,2] and briefly in Figure S1.

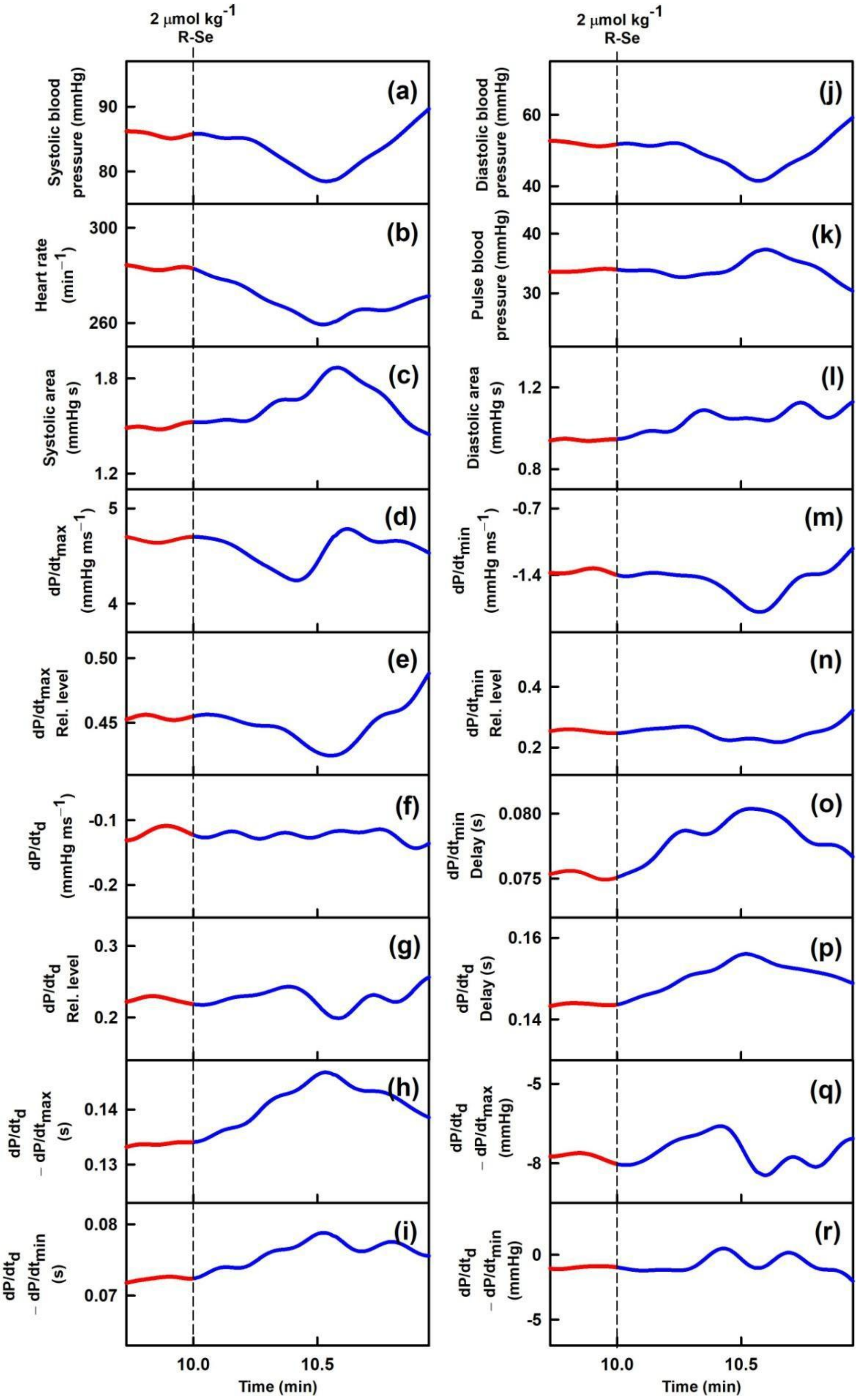

2 μmol kg-1
R-Se
(a)
Systolic blood pressure (mmHg)
(b)
Heart rate (min-1)
(c)
Systolic area (mmHg s)
(d)
dP/dtmax (mmHg ms-1)
(e)
dP/dtmax Rel. level
(f)
dP/dtd (mmHg ms-1)
(g)
dP/dtd Rel. level
(h)
dP/dtd − dP/dtmax (s)
(i)
dP/dtd − dP/dtmin (s)
(j)
Diastolic blood pressure (mmHg)
(k)
Pulse blood pressure (mmHg)
(l)
Diastolic area (mmHg s)
(m)
dP/dtmin (mmHg ms-1)
(n)
dP/dtmin Rel. level
(o)
dP/dtmin Delay (s)
(p)
dP/dtd Delay (s)
(q)
dP/dtd − dP/dtmax (mmHg)
(r)
dP/dtd − dP/dtmin (mmHg)
Time (min)

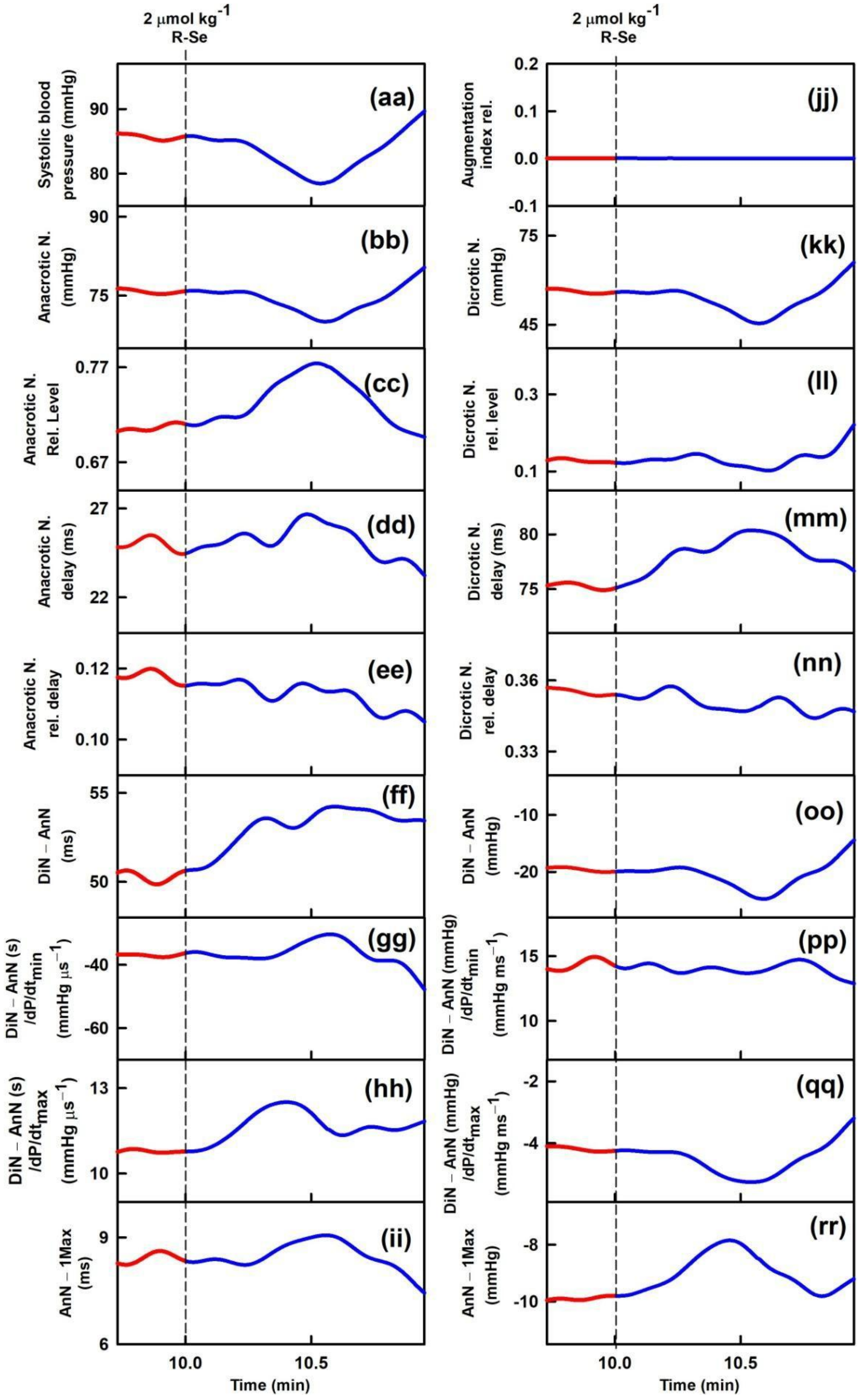

Figure S5. Time-dependent changes of 35 APW-Ps of anaesthetized rat before (red) and after IV bolus (15 s) administration of 2 μmol kg^{-1} R-Se (blue). Vertical dash lines indicate start of R-Se administration. Definitions, units and abbreviations of APW-Ps evaluated from the APW are explained in [1,2] and briefly in Figure S1.

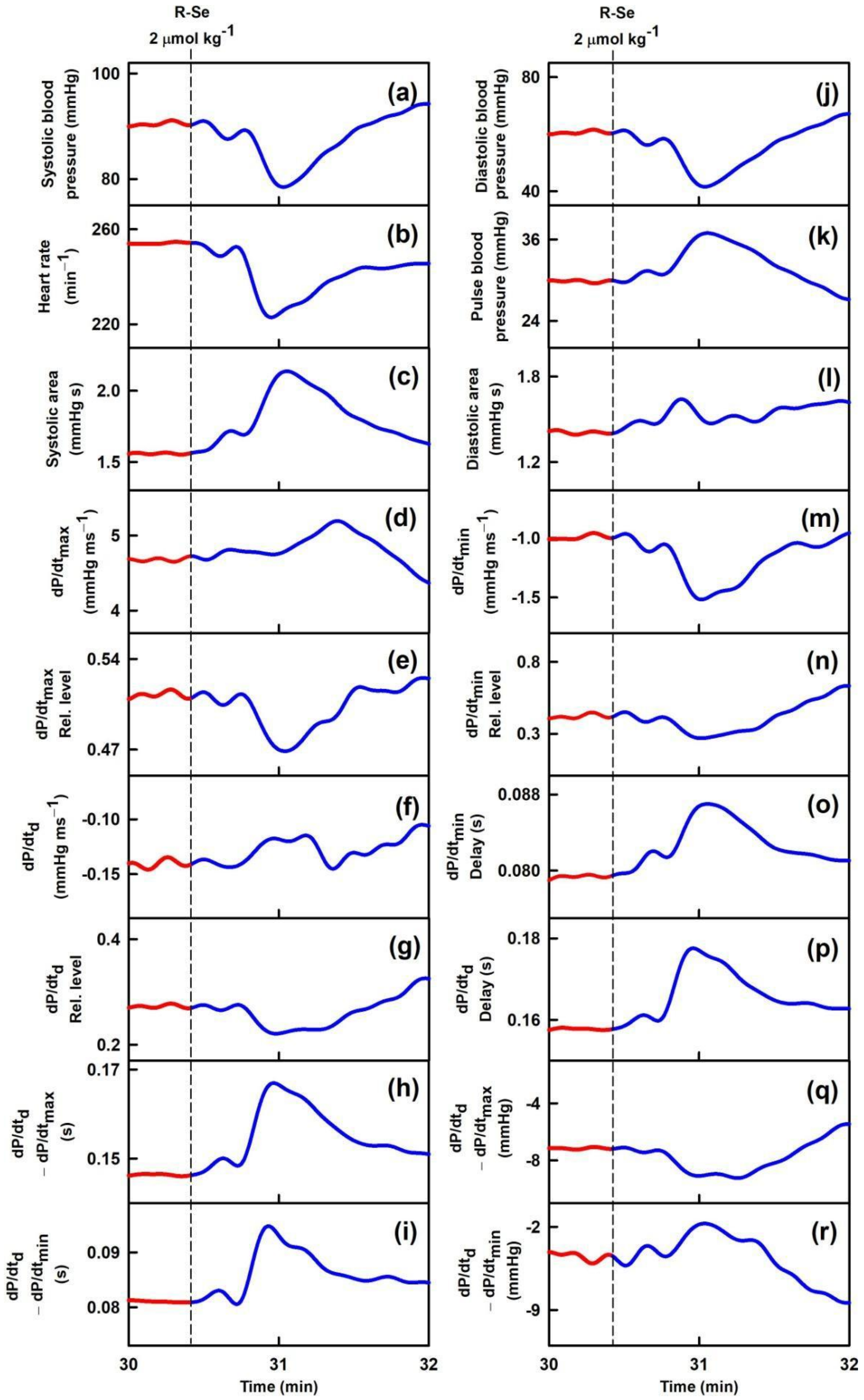
R-Se
2 μmol kg-1
Systolic blood pressure (mmHg)
Heart rate (min-1)
Systolic area (mmHg s)
dP/dtmax (mmHg ms-1)
dP/dtmax Rel. level
dP/dtd (mmHg ms-1)
dP/dtd Rel. level
dP/dtd − dP/dtmax (s)
dP/dtd − dP/dtmin (s)
Diastolic blood pressure (mmHg)
Pulse blood pressure (mmHg)
Diastolic area (mmHg s)
dP/dtmin (mmHg ms-1)
dP/dtmin Rel. level
dP/dtmin Delay (s)
dP/dtd Delay (s)
dP/dtd − dP/dtmax (mmHg)
dP/dtd − dP/dtmin (mmHg)
(a)
(b)
(c)
(d)
(e)
(f)
(g)
(h)
(i)
(j)
(k)
(l)
(m)
(n)
(o)
(p)
(q)
(r)
Time (min)

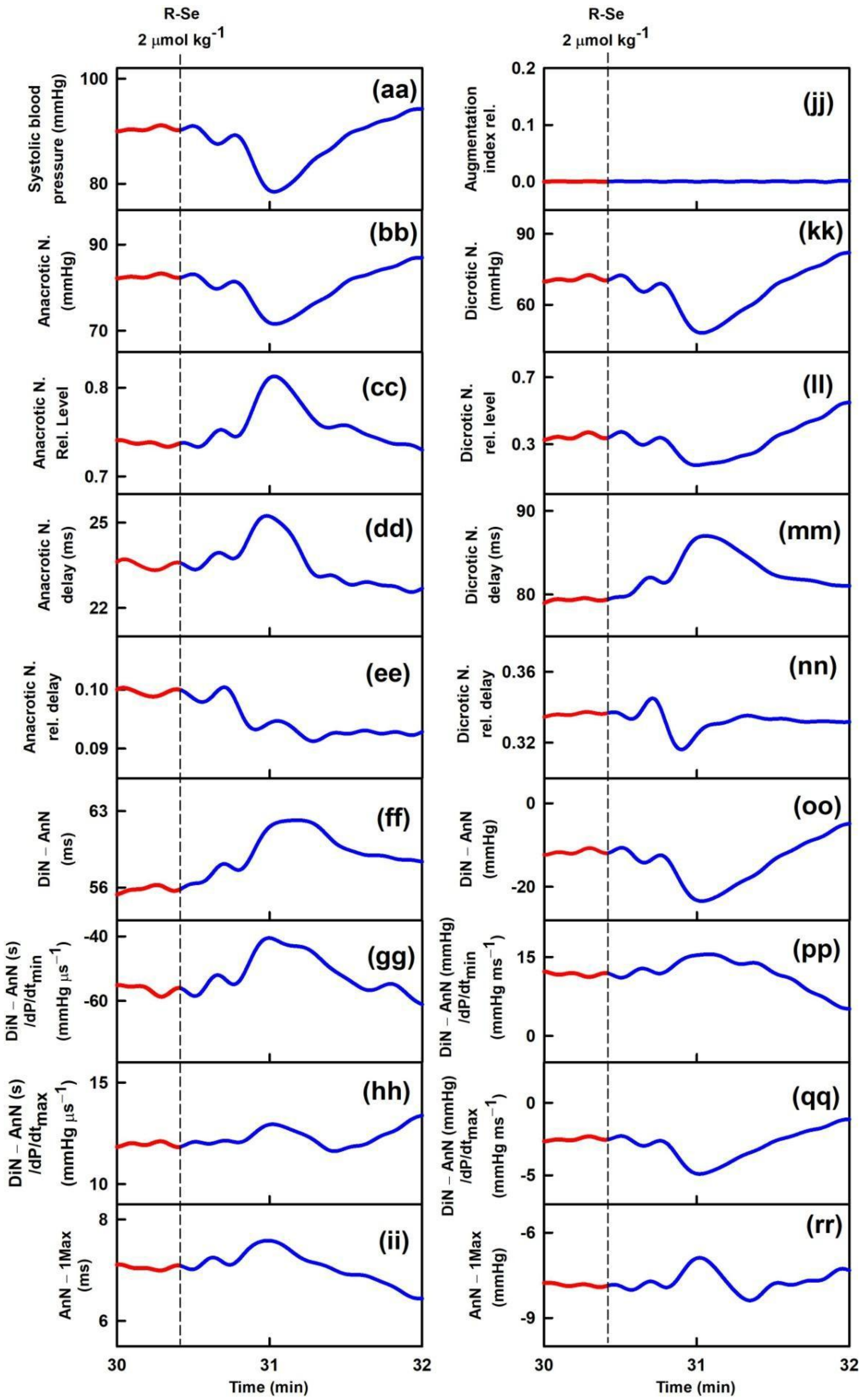

Figure S6. Time-dependent changes of 35 APW-Ps of anaesthetized rat before (red) and after IV bolus (15 s) administration of 2 μmol kg^{-1} R-Se (blue). Vertical dash lines indicate start of R-Se administration. Definitions, units and abbreviations of APW-Ps evaluated from the APW are explained in [1,2] and briefly in Figure S1.

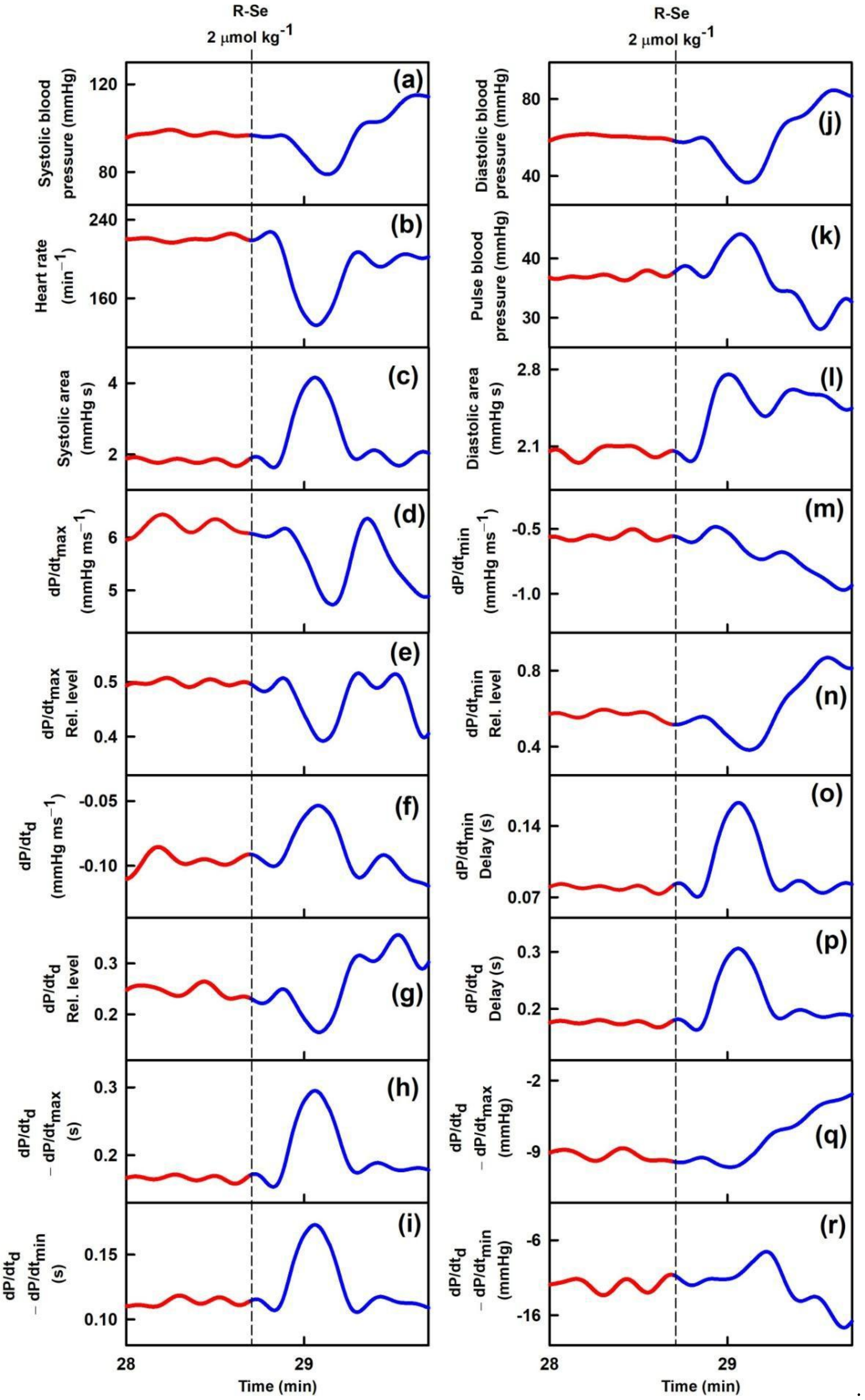
R-Se
2 μmol kg-1
Systolic blood pressure (mmHg)
Heart rate (min-1)
Systolic area (mmHg s)
dP/dtmax (mmHg ms-1)
dP/dtmax Rel. level
dP/dtd (mmHg ms-1)
dP/dtd Rel. level
dP/dtd − dP/dtmax (s)
dP/dtd − dP/dtmin (s)
Diastolic blood pressure (mmHg)
Pulse blood pressure (mmHg)
Diastolic area (mmHg s)
dP/dtmin (mmHg ms-1)
dP/dtmin Rel. level
dP/dtmin Delay (s)
dP/dtd Delay (s)
dP/dtd − dP/dtmax (mmHg)
dP/dtd − dP/dtmin (mmHg)
(a)
(b)
(c)
(d)
(e)
(f)
(g)
(h)
(i)
(j)
(k)
(l)
(m)
(n)
(o)
(p)
(q)
(r)
Time (min)

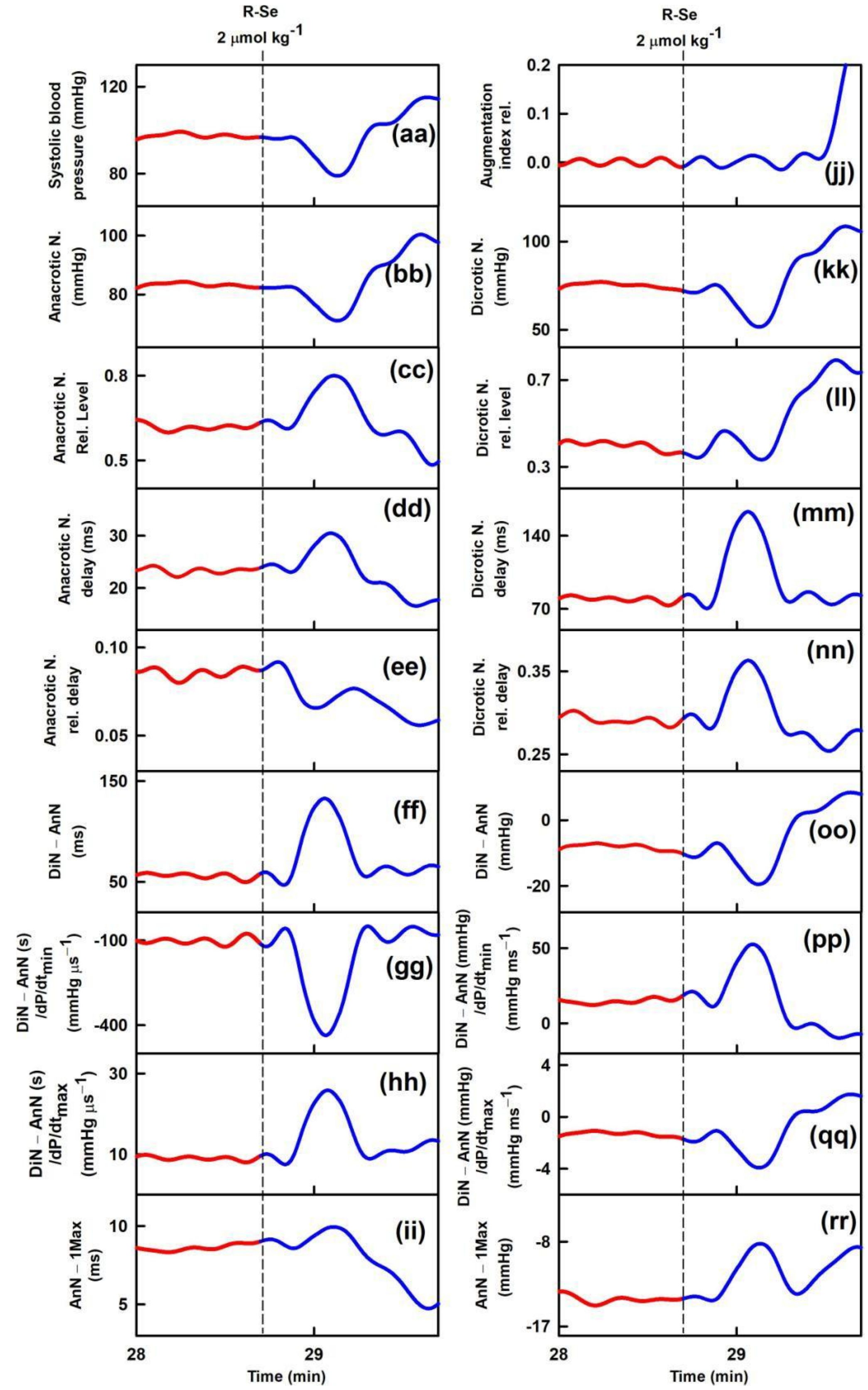

Figure S7. Time-dependent changes of 35 APW-Ps of anaesthetized rat before (red) and after IV bolus (15 s) administration of 2 μmol kg^{-1} R-Se (blue). Vertical dash lines indicate start of R-Se administration. Definitions, units and abbreviations of APW-Ps evaluated from the APW are explained in [1,2] and briefly in Figure S1.

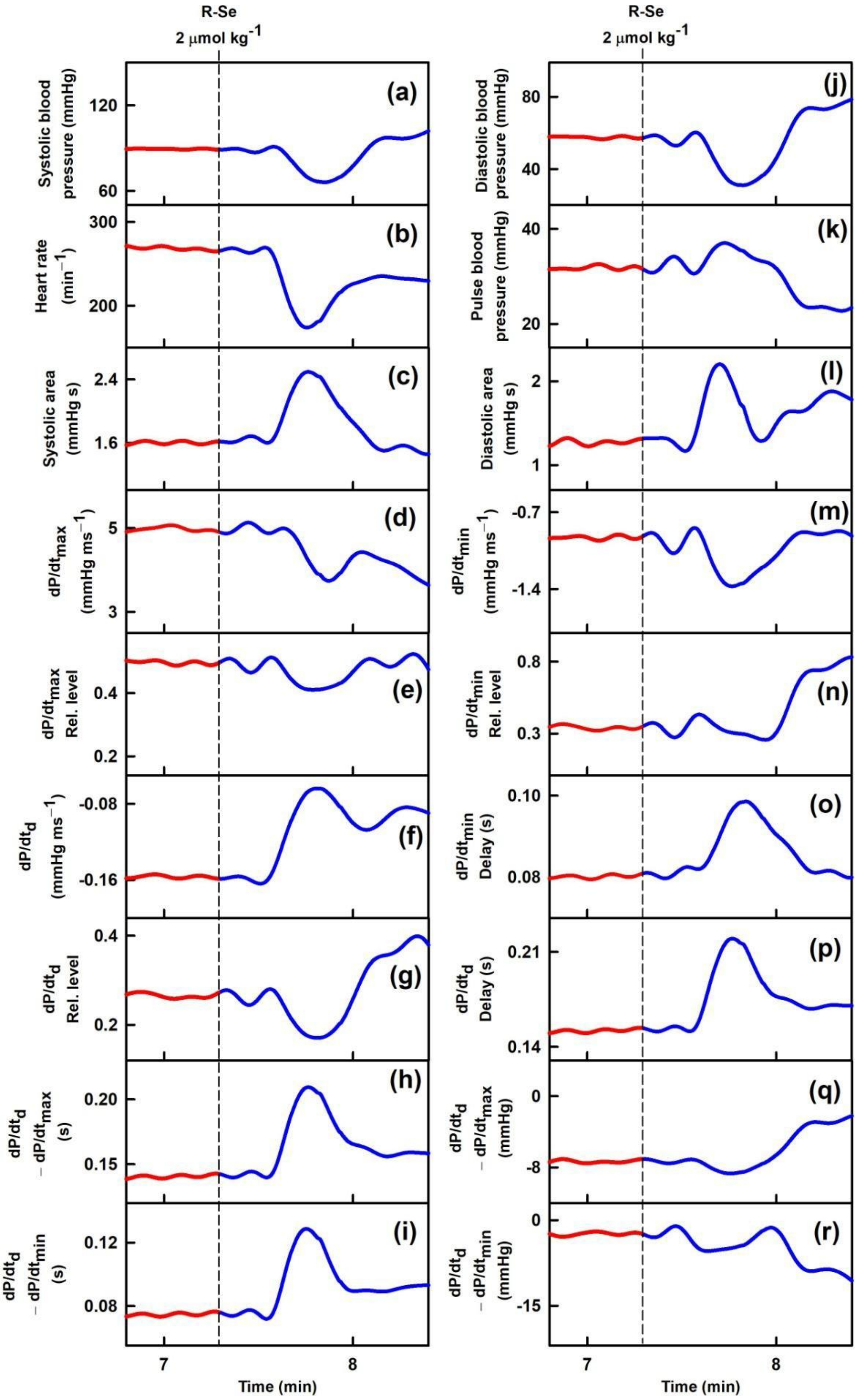

R-Se
2 μmol kg⁻¹
Systolic blood pressure (mmHg)
Heart rate (min⁻¹)
Systolic area (mmHg s)
dP/dt_max (mmHg ms⁻¹)
dP/dt_max Rel. level
dP/dt_d (mmHg ms⁻¹)
dP/dt_d Rel. level
dP/dt_d − dP/dt_max (s)
dP/dt_d − dP/dt_min (s)
Diastolic blood pressure (mmHg)
Pulse blood pressure (mmHg)
Diastolic area (mmHg s)
dP/dt_min (mmHg ms⁻¹)
dP/dt_min Rel. level
dP/dt_min Delay (s)
dP/dt_d Delay (s)
dP/dt_d − dP/dt_max (mmHg)
dP/dt_d − dP/dt_min (mmHg)
(a) (b) (c) (d) (e) (f) (g) (h) (i)
(j) (k) (l) (m) (n) (o) (p) (q) (r)
Time (min)

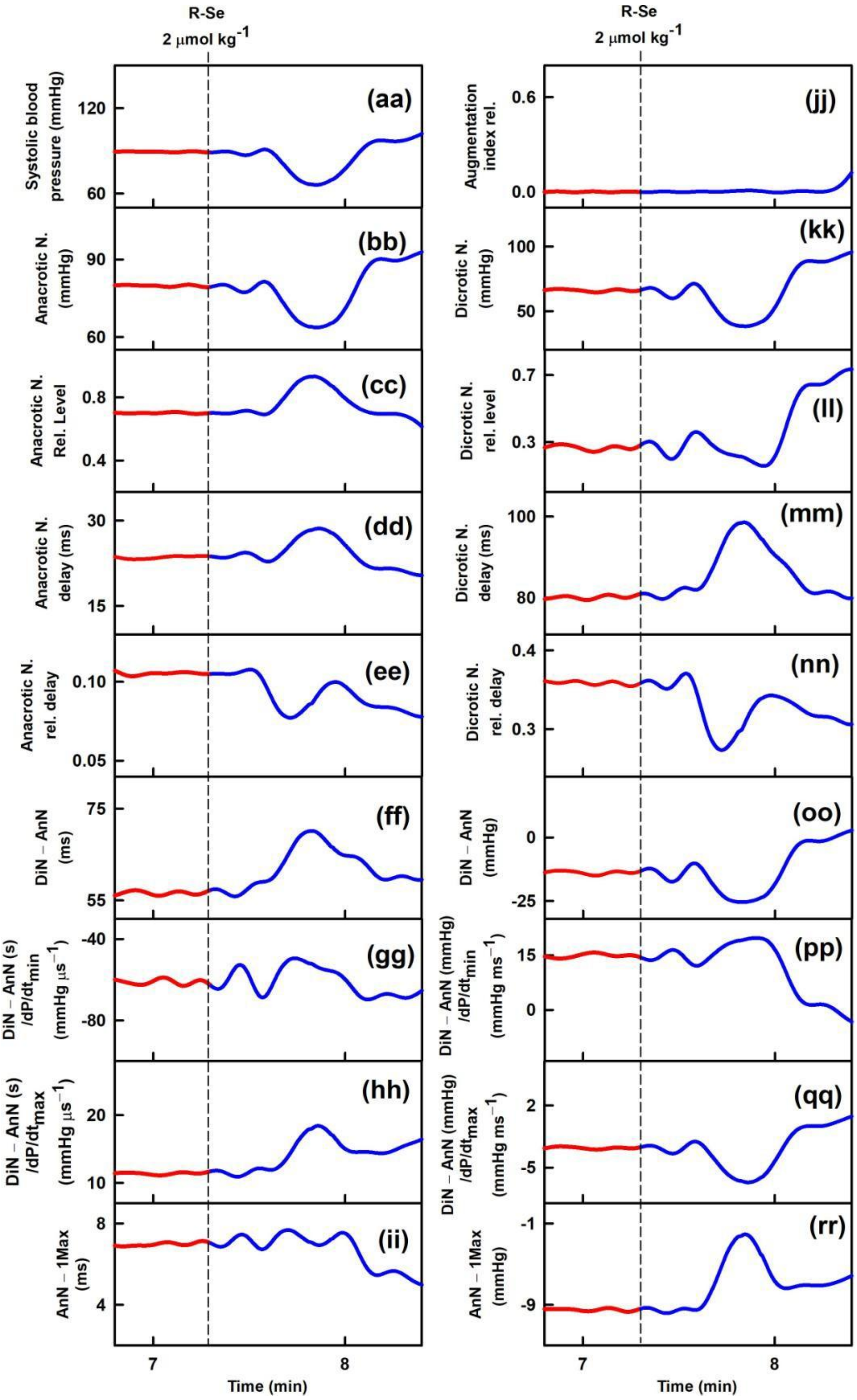

Figure S8. Time-dependent changes of 35 APW-Ps of anaesthetized rat before (red) and after IV bolus (15 s) administration of 2 μmol kg^{-1} R-Se (blue). Vertical dash lines indicate start of R-Se administration. Definitions, units and abbreviations of APW-Ps evaluated from the APW are explained in [1,2] and briefly in Figure S1.

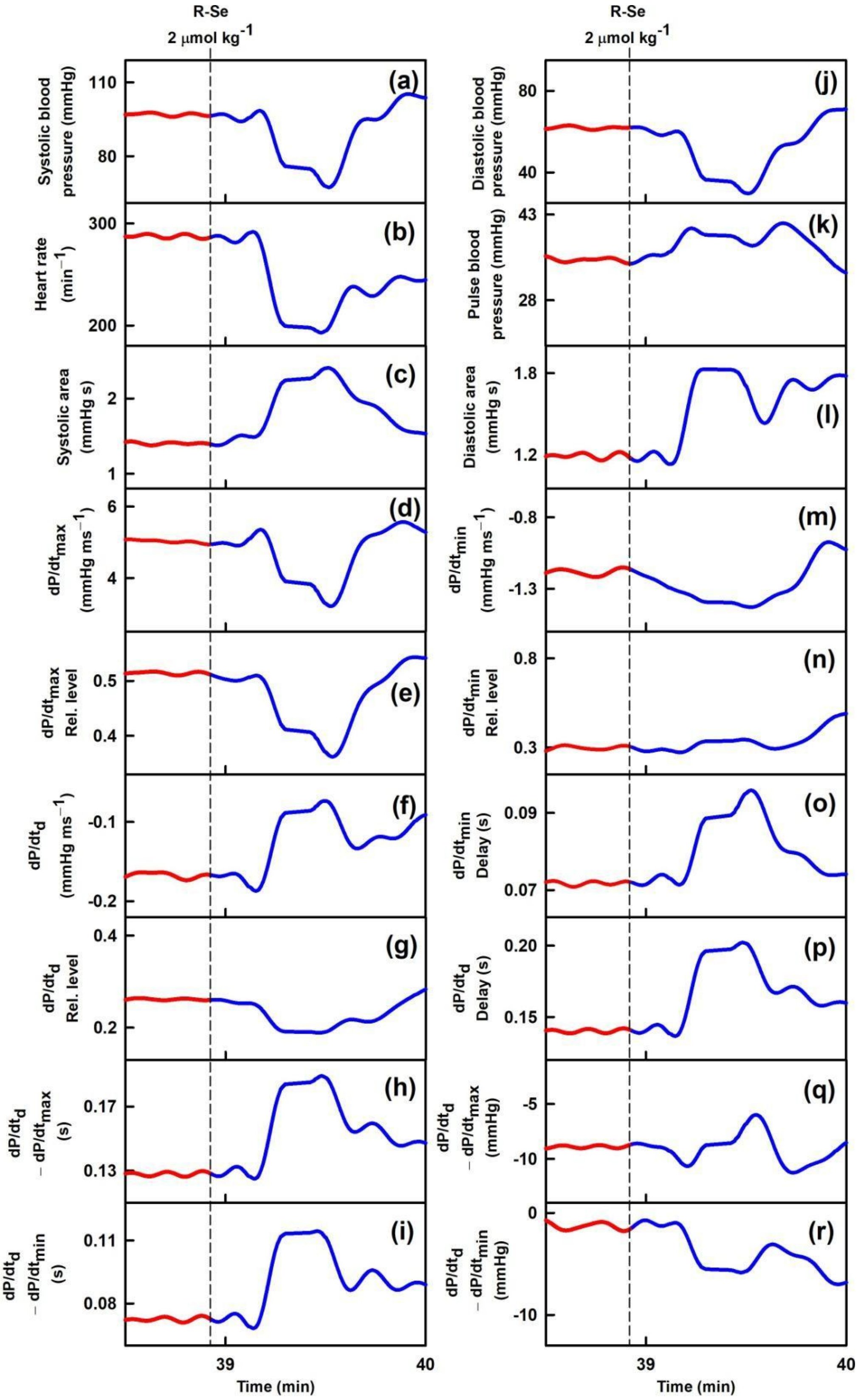
R-Se
2 μmol kg-1
Systolic blood pressure (mmHg)
Heart rate (min-1)
Systolic area (mmHg s)
dP/dtmax (mmHg ms-1)
dP/dtmax Rel. level
dP/dtd (mmHg ms-1)
dP/dtd Rel. level
dP/dtd – dP/dtmax (s)
dP/dtd – dP/dtmin (s)
Diastolic blood pressure (mmHg)
Pulse blood pressure (mmHg)
Diastolic area (mmHg s)
dP/dtmin (mmHg ms-1)
dP/dtmin Rel. level
dP/dtmin Delay (s)
dP/dtd Delay (s)
dP/dtd – dP/dtmax (mmHg)
dP/dtd – dP/dtmin (mmHg)
(a)
(b)
(c)
(d)
(e)
(f)
(g)
(h)
(i)
(j)
(k)
(l)
(m)
(n)
(o)
(p)
(q)
(r)
Time (min)

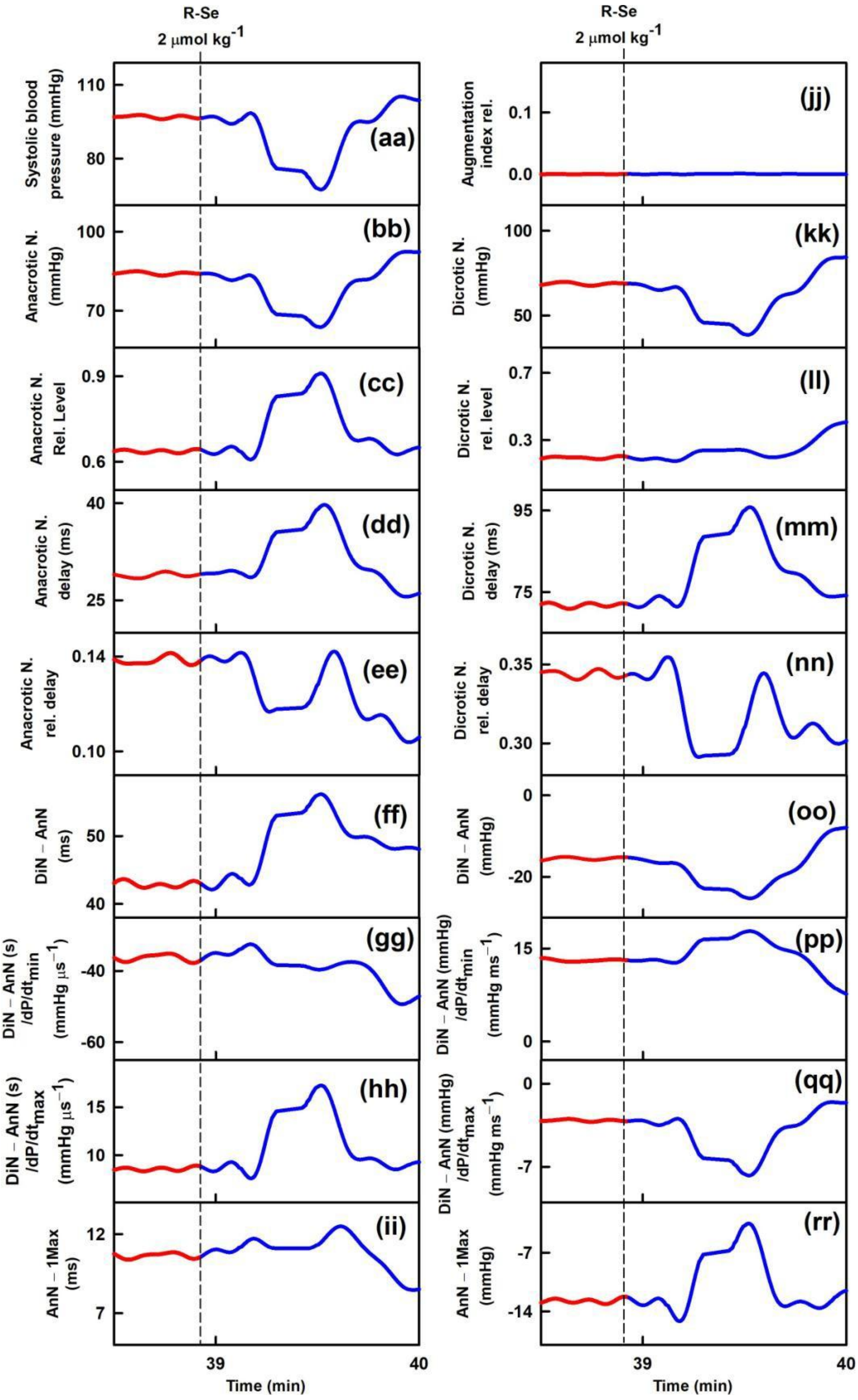

Figure S9. Time-dependent changes of 35 APW-Ps of anaesthetized rat before (red) and after IV bolus (15 s) administration of 2 μmol kg^{-1} R-Se (blue). Vertical dash lines indicate start of R-Se administration. Definitions, units and abbreviations of APW-Ps evaluated from the APW are explained in [1,2] and briefly in Figure S1.

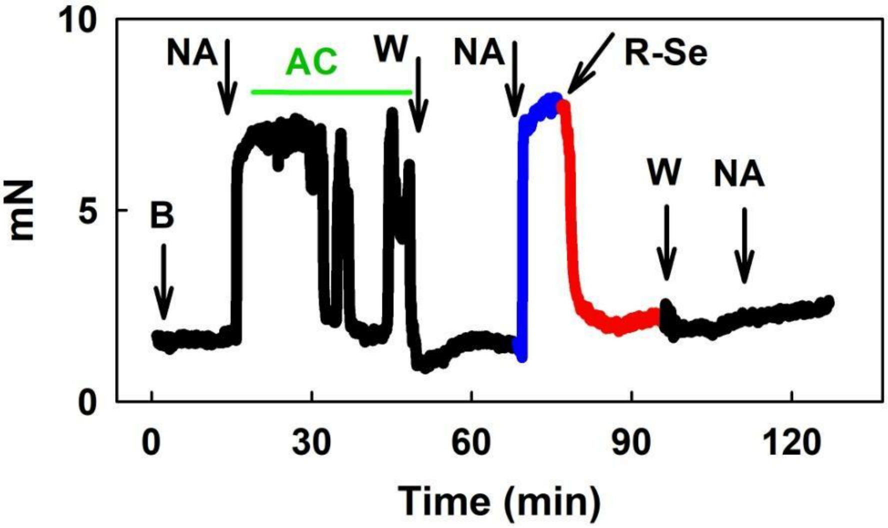

Figure S10. Original recording of the vascular reactivity of the mesenteric artery.
B indicates baseline. Vascular segment was pre-contracted with noradrenaline (**NA**, 10 μmol L^{-1}). Acetylcholine was added in a cumulative manner (**AC**, 1 nmol L^{-1} - 10 μmol L^{-1}). The vessel was washed four times (**W**) and re-contracted with **NA**. After stabilization of the tension, **R-Se** (12.5 μmol L^{-1}) was applied. The vessel was washed four times again (**W**) and **NA** (10 μmol L^{-1}) was applied.

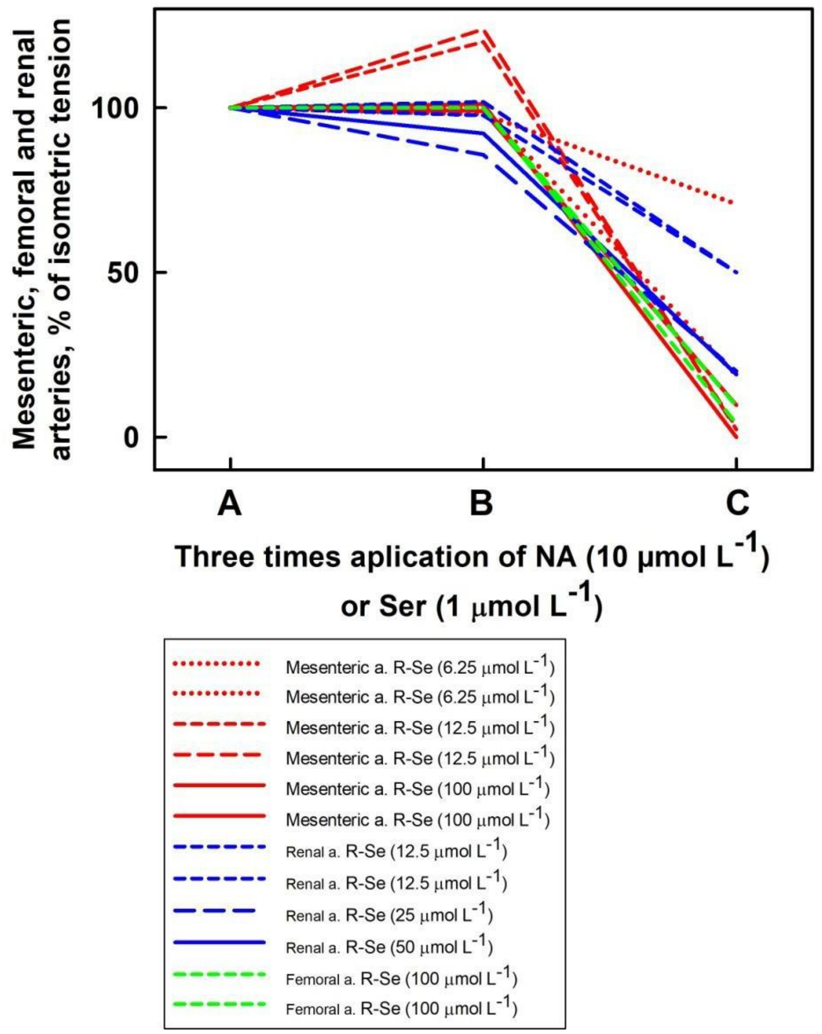

Figure S11. The comparison of noradrenaline (NA, 10 μmol L^{-1}) or serotonin (Ser, 1 μmol L^{-1}) potency to increase tension of the mesenteric, renal and femoral arteries during control measurement of vascular endothelium function (A), before application of R-Se (B) and after wash out of R-Se (C). For details see Figure S10.

References

1. Kurakova, L.; Misak, A.; Tomasova, L.; Cacanyiova, S.; Berenyiova, A.; Ondriasova, E.; Balis, P.; Grman, M.; Ondrias, K. Mathematical relationships of patterns of 35 rat haemodynamic parameters for conditions of hypertension resulting from decreased nitric oxide bioavailability. *Exp. Physiol.* **2020**, *105*, 312-334.
2. Tomasova, L.; Grman, M.; Misak, A.; Kurakova, L.; Ondriasova, E.; Ondrias, K. Cardiovascular "Patterns" of H_2S and $SSNO^-$-Mix Evaluated from 35 Rat Hemodynamic Parameters. *Biomolecules* **2021**, *11*, 293.
